# Matrix feedback enables diverse higher-order patterning of the extracellular matrix

**DOI:** 10.1371/journal.pcbi.1007251

**Published:** 2019-10-28

**Authors:** Esther Wershof, Danielle Park, Robert P. Jenkins, David J. Barry, Erik Sahai, Paul A. Bates

**Affiliations:** 1 Biomolecular Modelling Laboratory, The Francis Crick Institute, London, United Kingdom; 2 Tumour Cell Biology Laboratory, The Francis Crick Institute, London, United Kingdom; 3 Advanced Light Microscopy Facility, The Francis Crick Institute, London, United Kingdom; Oxford, UNITED KINGDOM

## Abstract

The higher-order patterning of extra-cellular matrix in normal and pathological tissues has profound consequences on tissue function. Whilst studies have documented both how fibroblasts create and maintain individual matrix fibers and how cell migration is altered by the fibers they interact with, a model unifying these two aspects of tissue organization is lacking. Here we use computational modelling to understand the effect of this interconnectivity between fibroblasts and matrix at the mesoscale level. We created a unique adaptation to the Vicsek flocking model to include feedback from a second layer representing the matrix, and use experimentation to parameterize our model and validate model-driven hypotheses. Our two-layer model demonstrates that feedback between fibroblasts and matrix increases matrix diversity creating higher-order patterns. The model can quantitatively recapitulate matrix patterns of tissues *in vivo*. Cells follow matrix fibers irrespective of when the matrix fibers were deposited, resulting in feedback with the matrix acting as temporal ‘memory’ to collective behaviour, which creates diversity in topology. We also establish conditions under which matrix can be remodelled from one pattern to another. Our model elucidates how simple rules defining fibroblast-matrix interactions are sufficient to generate complex tissue patterns.

## Author summary

The organization of extracellular matrix in the body is diverse and often becomes dysregulated in cancer. How such patterns emerge is poorly understood, in particular how the interplay between the fibroblasts, which produce the matrix fibers, and the fibers themselves affects emergent organization. We created a unique adaptation to the Vicsek flocking model to include feedback from a second layer representing the matrix. Fibroblasts were able to deposit, degrade and rearrange fibers and the fibers could also contribute to the vector of cell migration. We find that this feedback is sufficient to generate many complex tissue patterns, as seen *in vivo*. The model is able to encapsulate key fibroblast and matrix properties without compromising on scale, providing an insight into the co-evolution of two crucial components of tissues at the scale of millimeters.

## Introduction

The extracellular matrix (ECM) provides support and enables the functioning of tissues within multicellular organisms [1, 2]. Depending on the tissue, the ECM can be organized in a myriad of different ways [3–5] from highly aligned linear bundles in connective tissue such as tendons, to largely anisotropic meshwork in basement membranes [6]. Curved bundles of ECM fibers are found in mammary tissue that can be loose, diffuse curls or tightly coiled bundles, for example in the dermis [7]. The patterning of ECM has large consequences on tissue function and often becomes disregulated in disease. How these different topologies emerge and subsequently interconvert as organisms age and develop pathologies, such as cancer, remains largely unknown. ECM is produced by a variety of different cells, with fibroblasts playing a particularly prominent role [8–11]. Numerous studies have documented the biosynthetic pathways of ECM production and some of the principles of fiber assembly [12, 13]. However, the relationship between cell behavior and the emergent patterns of matrix organization at the mesoscale (millimeter scale) is unclear. The problem is further complicated because cell behavior is instructed by the ECM [14, 15], with cells aligning themselves with matrix fibers and responding to different matrix stiffness with altered cell signaling and cell migration [1, 16, 17]. Mechanoreciprocity and mechanotransduction have been shown to be crucial phenomena in determining tissue and tumor evolution [18, 19]. The feedback between cell behavior and matrix organization therefore presents a ‘chicken and egg’ problem when trying to understand the development of higher order patterns. Does matrix structure exert a dominant role over cell behavior that then reinforces the matrix topology? Or does cell behavior initiate matrix patterning? The lack of matrix at the zygote stage of development suggests that cell behavior is somehow the initial determinant of matrix patterning, but it does not preclude an important role for feedback from the extracellular matrix in specifying pattern formation.

Fibroblasts are largely responsible for producing, degrading and rearranging matrix components and enabling their assembly into larger complex structures. This latter process is highly dependent on the integrin family of matrix receptors that span the plasma membrane and connect the cell’s cytoskeleton to the extra-cellular matrix. Many perturbations have been identified that lead to altered matrix anisotropy, including within integrins, integrin-associated molecules, and the actin cytoskeleton, generating simple patterns of alignment and disorder. There have been numerous studies documenting the diversity of fibroblast behavior [20, 21] and the ability of cells to cooperate, often across many orders of magnitude [22–26]. Yet it remains unclear how complex tissue patterns develop and why particular perturbations alter such patterns. Tackling this problem experimentally is challenging due to the complexity of systems with many cells and matrix components, and the difficulty of seeing in real time how matrix is being organized. To understand how specific rules defining cell-matrix interactions can generate emergent behaviors, we therefore turn to computational modeling.

Modeling of such behavior requires the inclusion of discrete fibers, rendering continuum models less appropriate. A Cellular Potts model has been used in a previous study to explore cell migration in ECM [27]; however, it is difficult to model explicit fibers to behave in a physiologically reasonable way and to implement rules for cell adhesion specifically between the cells and individual fibers without making the system far more complicated. Simple models of nematically aligning self-propelled rods [28–30] serve as a good basis for modeling collective cell behavior but move in a continuum fluid and have a simplistic averaging for coordination between rods. Hybrid models with discrete fibroblasts and a continuum matrix have been used to explore scarring in wound healing [31, 32] and fibroblast alignment [33]. Work by Dallon et al. [34] explores interactions between the cells and matrix. They report that matrix feedback can reduce overall alignment, which we confirm here. The authors also show that a strip of aligned fibers that is sufficiently thick can cause the rest of the matrix to align similarly. Our work builds on such hybrid models by incorporating cell-cell interactions and cell migratory noise explicitly. Further, we model fibers discretely, allowing for an understanding of how an individual fiber can influence cell motility.

The Vicsek model [35] has been used for many decades to explore collective behavior of animals at high density, most notably shoals of fish and flocks of birds. In this approach, migration of animals or cells is simulated with individual velocities. Crucially, the vector of migration is determined by the combination of the level of intrinsic persistence [36] of a cell (influenced by the magnitude of a Gaussian noise term [37]), henceforth called individual migratory noise, and the propensity for a cell to align its directionality with that of nearby individuals. Within certain regions of parameter space this can generate rapid and effective alignment. Several extensions of the Vicsek model and models with similar neighbor alignment [29, 38, 39] or velocity alignment [40–42] have been developed to tackle specific biological questions [43], for example, how *Dictyoselium discoideum* can aggregate as a result of chemotaxis towards pulses of cyclic adenosine monophosphate and when this switch to collective behaviour occurs [44]. However, in its simple form, the Vicsek model is not well suited to modelling the interplay between cells and ECM. Matrix persists over long time scales and can be both synthesized and degraded, whereas the alignment term in the Vicsek model only considers the vector of individuals spatially local at the current time step. To circumvent this, we have developed a two-layer adaptation of the Vicsek model. One layer represents migratory cells and is similar to the conventional Vicsek model, the second represents the matrix and records the cumulative effect of cells on matrix synthesis and degradation. This second layer contributes an additional term in the behavior of the cells that represents matrix feedback on cell behavior. By exploiting this model in combination with experimentation we explore the role of matrix feedback in the generation of higher order patterns of ECM.

## Results

### Diverse matrix patterns are found *in vivo* and can be quantified

**Fig 1:**
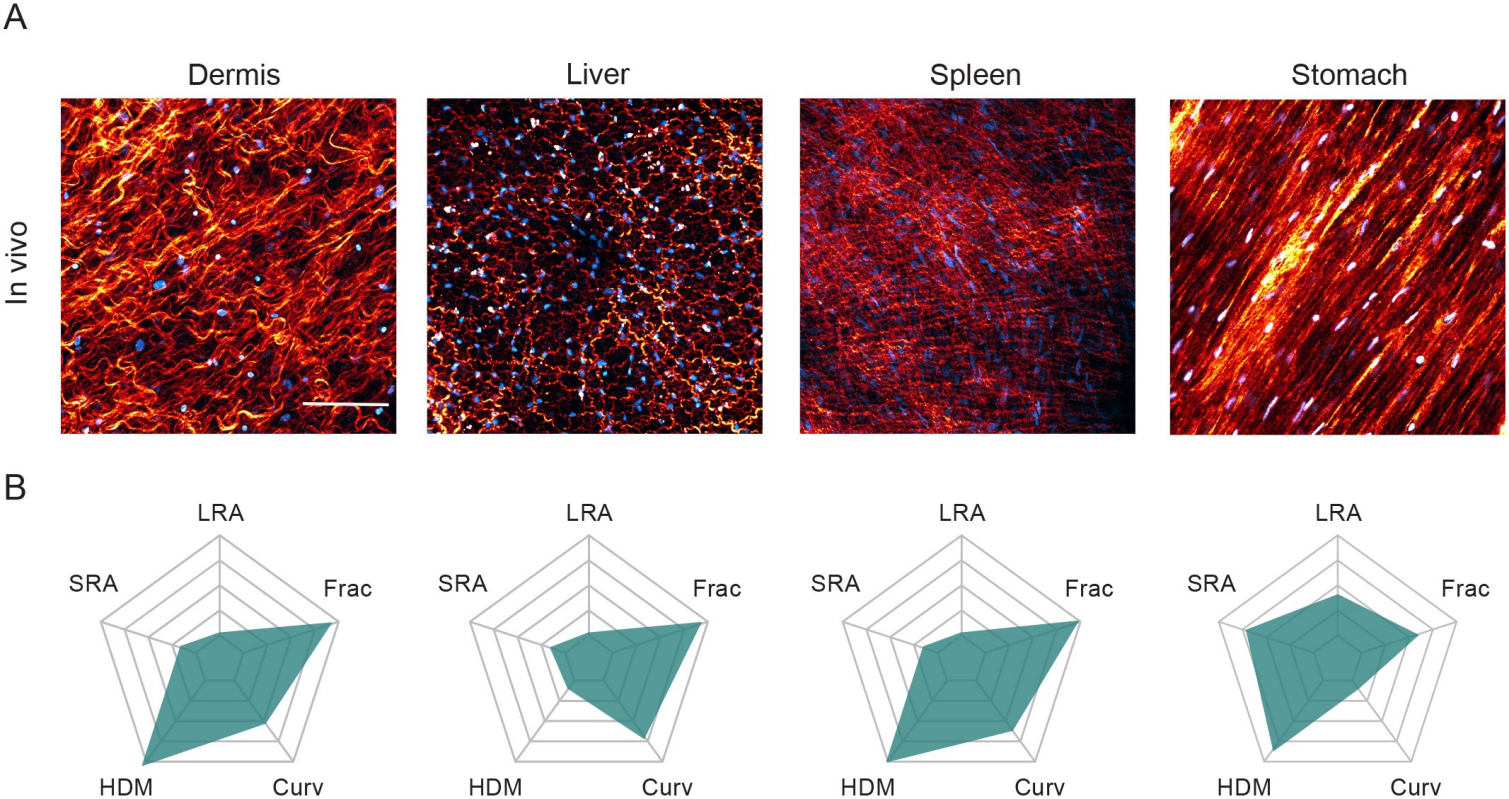
The ECM exhibits diversity *in vivo*. (A) Second harmonic imaging of collagen (orange) in various tissues from a PDGFR nuclear labelled mouse. PDGFR positive nuclei (cyan) indicates fibroblast density. (B) Each matrix is characterized on a starplot below the image according to long-range alignment (LRA), short-range alignment (SRA), percentage of high density matrix (HDM), curvature (Curv) and fractal dimension (Frac). Scale bars represent 100*μ*m.

Second harmonic imaging of collagen fibers in different mouse tissues *in vivo* revealed various patterns of matrix organization (Fig 1a). The position of fibroblasts was determined by using mice with transgenic expression of a fluorescent nuclear marker, H2B-GFP, in fibroblasts. Of note, the long axis of the nucleus indicates the orientation of the fibroblast cell body and a clear correlation between fibroblast and matrix alignment is visible in stomach ECM and has been previously documented [5].

To quantify the matrix diversity observed *in vivo*, we derived five metrics of matrix organization: long-range alignment of fibers (LRA), short-range alignment (SRA) (S1a Fig), percentage of high density matrix (HDM) (S1b Fig), curvature (Curv) (S1c Fig) and fractal dimension (Frac) (S1d Fig). Collectively, these metrics can describe a range of matrix properties. For example, a swirl-like matrix has low long-range alignment whilst maintaining some short-range alignment (dermis, Fig 1a). Such a matrix would also have high curvature. The percentage of high density matrix encapsulates how homogeneous a matrix is. If cells are corralled, hence channeled to cover specific regions, this would result in more high-density matrix (liver, Fig 1a). Fractal dimension measures the self-similarity of the matrix and the extent to which the one-dimensional fibers can fill two-dimensional space [45]. A diffuse matrix will have a higher fractal dimension than matrix with large gaps and a non-aligned matrix, with fibers oriented in many different directions will have a higher fractal dimension than an aligned matrix, for example the spleen as compared to the stomach in Fig 1a. Examples of these metrics are given in S1 Fig. Application of these metrics to both murine and human pathological matrix confirms the diversity of matrix organization. For easy visualization of the different metrics, we generated star plots with five axes, each reflecting a different metric (Fig 1b).

### Model description

**Fig 2:**
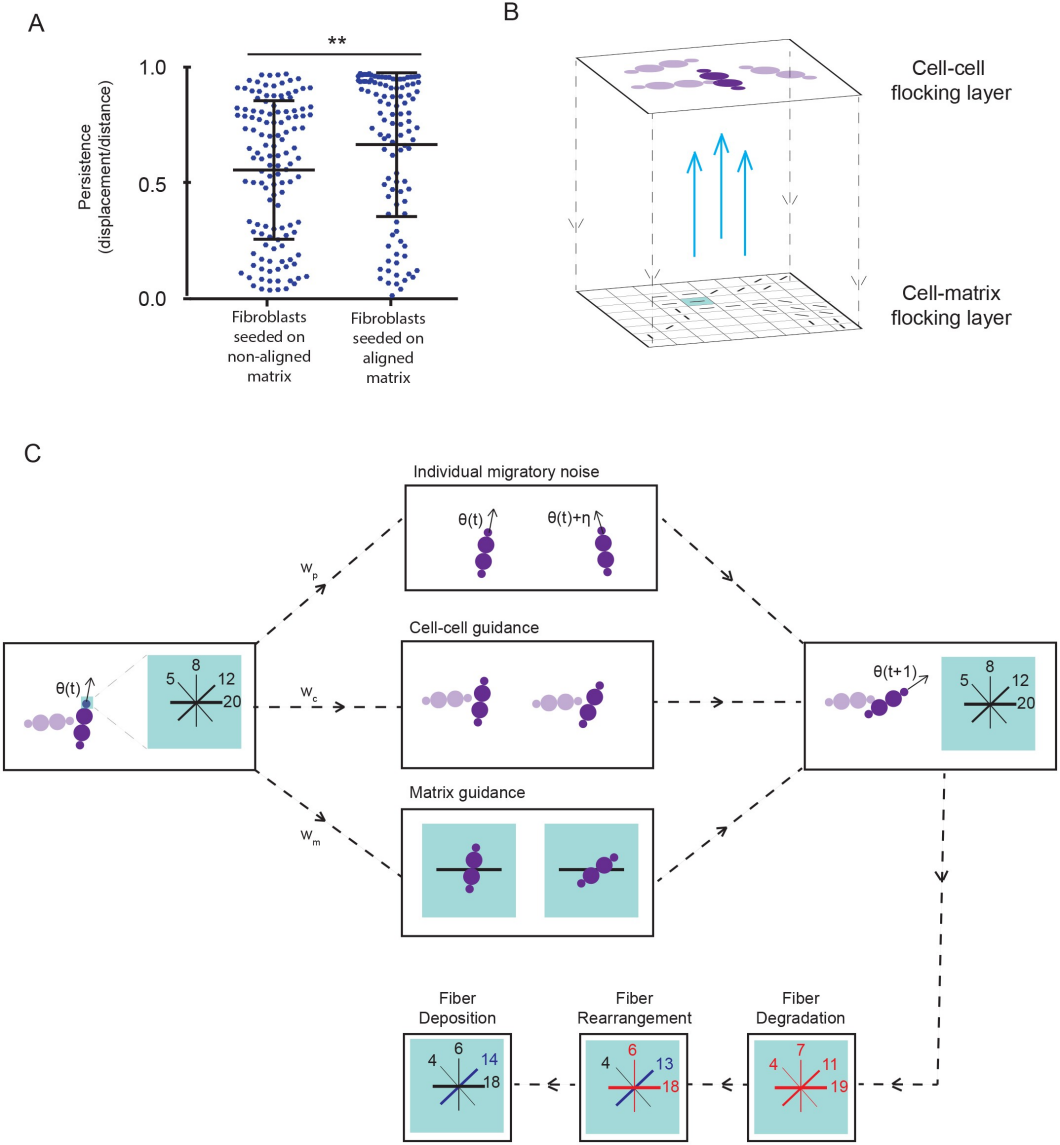
A multi-layered flocking model of cell-cell and cell-matrix interactions. (A) Migratory persistence of aligning or non-aligning fibroblasts seeded on a thick aligned matrix (over 8 hour windows). On an aligned matrix, fibroblasts migrate with higher persistence (p = 0.0064, using a two-tailed t-test). (B) Schematic showing the two-layer set up of the model. (C) Schematic describing the model. Cells flock with other cells they come into contact with and the fibers in the matrix grid point underneath the head of the cell. At every time step, for each cell in turn we compute its change in orientation due to flocking with other cells, its change in orientation due to flocking with the matrix below and its reorganization of the matrix in the grid point below via degradation, rearrangement and deposition of fibers. In this schematic, there are four bins per grid point for fibers to be deposited in.

To explore the mechanisms by which fibroblasts generate matrix patterns and how divergent matrix topologies initially arise, we initially adapted the Vicsek flocking model with cells moving subject to individual migratory noise and influence by their local neighbors [35]. A fibroblast’s individual migratory noise reflects its persistent migration as a result of cell polarization [46]. At time zero, cells were placed at random and each cell assigned a random orientation (Fig 2) and a constant speed drawn from a Gaussian distribution. The change in the orientation of a cell is computed as a weighted function of three terms: individual migratory noise, cell-cell guidance and matrix guidance. Unlike the original Vicsek model, cells were able to coordinate their behavior nematically [28] and the extent of coordination could be explicitly controlled by the cell-cell guidance term. Fibroblasts are modelled as a bead representing the head of the cell, followed by two beads of twice the diameter representing the cell body, followed by another smaller bead for the tail. This diamond-like shape has aspect ratio 1:3 and reflects a typical cell morphology [47]. For a cell *i* at time *t*, *θ*_*i*_(*t*) denotes its orientation, *s*_*i*_ denotes its speed and (*x*_*i*_(*t*), *y*_*i*_(*t*)) denotes its position.

#### Individual migratory noise

Cells move along their long axis at a constant speed and the individual migratory noise of a cell is modelled as a persistent random walk *θ*_*i*_(*t*) + *η*_*i*_(*t*), where *η*_*i*_(*t*) is Gaussian distributed with mean zero. The positional X and Y-components of this term can be defined as

$$\begin{array}{c} X_{p}=cos\left(\theta_{i}\left(t\right)+\eta_{i}\left(t\right)\right). \end{array}$$ (1)

$$\begin{array}{c} Y_{p}=sin\left(\theta_{i}\left(t\right)+\eta_{i}\left(t\right)\right). \end{array}$$ (2)

#### Cell-cell guidance

If a cell *i* is in direct contact with *N* cells, *N* > 0, then the X and Y-components of the effect of cell guidance on *i* are defined as

$$\begin{array}{c} X_{c}=\frac{1}{N}\sum_{j=1}^{N}cos\left(\tilde{\theta_{j}}\left(t\right)\right), \end{array}$$ (3)

$$\begin{array}{c} Y_{c}=\frac{1}{N}\sum_{j=1}^{N}sin\left(\tilde{\theta_{j}}\left(t\right)\right), \end{array}$$ (4)

where

$$\begin{array}{c} \tilde{\theta_{j}}\left(t\right)=\{\begin{array}{cc} \theta_{j}\left(t\right) & \;\text{if}\;|\theta_{j}\left(t\right)-\theta_{i}\left(t\right)|(\text{mod}\;\pi )<\frac{\pi }{2},\\ \theta_{j}\left(t\right)+\frac{\pi }{2} & \;\text{otherwise.} \end{array} \end{array}$$

This adaptation constitutes the ability of fibroblasts to align in a nematic manner. For greater tractability, an assumption of the model is that *w*_*c*_ remains fixed for any non-zero value of *N*, so that, provided a cell is in contact with at least one other cell, the degree of flocking will be fixed. This means cell-cell guidance is independent of *N* for *N* > 0. The angle of cell-cell flocking is an average of all *N* of a cell’s contacts. However, the confluence levels both *in silico* and *in vivo* mean that *N* is kept small in practice.

#### Matrix guidance

If the head of a cell *i* is above a fiber *k* with orientation *ϕ*_*k*_, then the X and Y-component of the effect of matrix guidance of *k* on *i* are

$$\begin{array}{c} X_{m}=cos\left(\tilde{\phi_{k}}\left(t\right)\right), \end{array}$$ (5)

$$\begin{array}{c} Y_{m}=sin\left(\tilde{\phi_{k}}\left(t\right)\right), \end{array}$$ (6)

where

$$\begin{array}{c} \tilde{\phi_{k}}\left(t\right)=\{\begin{array}{cc} \phi_{k}\left(t\right) & \;\text{if}\;|\phi_{k}\left(t\right)-\theta_{i}\left(t\right)|(\text{mod}\;\pi )<\frac{\pi }{2},\\ \phi_{k}\left(t\right)+\frac{\pi }{2} & \;\text{otherwise.} \end{array} \end{array}$$

representing the ability of fibroblasts to move along fibers in a nematic manner.

The X and Y-components of cell *i* are then written as a weighted function of the three terms:

$$\begin{array}{c} X_{i}=\frac{1}{w_{p}+w_{c}+w_{m}}\left(w_{p}X_{p}+w_{c}X_{c}+w_{m}X_{m}\right), \end{array}$$ (7)

$$\begin{array}{c} Y_{i}=\frac{1}{w_{p}+w_{c}+w_{m}}\left(w_{p}Y_{p}+w_{c}Y_{c}+w_{m}Y_{m}\right), \end{array}$$ (8)

where *w*_*p*_ = 1 − *w*_*c*_ − *w*_*m*_, 0 ≤ *w*_*p*_, *w*_*c*_, *w*_*m*_ ≤ 1. If a cell is not in contact with any other cell, or above any fiber then *w*_*c*_ or *w*_*m*_ respectively are set to zero. The orientation of cell *i* at time *t* + 1 is then computed as

$$\begin{array}{c} \theta_{i}\left(t+1\right)=tan^{-1}(\frac{Y_{i}}{X_{i}}), \end{array}$$ (9)

which must then be adjusted for quadrant of the arctan function so that

$$\begin{array}{c} \theta_{i}\left(t+1\right)=\{\begin{array}{cc} \theta_{i}\left(t+1\right) & \;\text{if}\;X_{i}\geq 0,Y_{i}\geq 0,\\ \theta_{i}\left(t+1\right)+\pi & \;\text{if}\;X_{i}<0,\\ \theta_{i}\left(t+1\right)+2\pi & \;\text{if}\;X_{i}\geq 0,Y_{i}<0. \end{array} \end{array}$$

Finally cell position is updated so that

$$\left[\begin{array}{c} x_{i}(t+1)\\ y_{i}(t+1) \end{array}\right]=\left[\begin{array}{c} x_{i}(t)+s_{i}cos(\theta_{i}(t+1))v_{e}\\ y_{i}(t)+s_{i}sin(\theta_{i}(t+1))v_{e} \end{array}\right]$$ (10)

where *v*_*e*_ is a proxy for volume exclusion defined by

$$\begin{array}{c} v_{e}=\{\begin{array}{cc} 0.25 & \;\text{if}\;\text{the}\;\text{head}\;\text{bead}\;\text{of}\;\text{a}\;\text{cell}\;\text{is}\;\text{overlapping}\;\text{with}\;\text{any}\;\text{other}\;\text{bead}\;\text{of}\;\text{another}\;\text{cell,}\\ 1 & \;\text{otherwise} \end{array} \end{array}$$

The distance between cells which is considered as an overlap is set by the user but for this work was set as the inner 75% of the cell’s area.

#### Matrix updates

We modified the Vicsek model to include a second layer, consisting of a grid of matrix fibers. This meant the model comprised two layers: a top layer of fibroblasts, and an underlying layer of extra-cellular matrix. The ECM layer was arranged in a grid and each grid point was associated with matrix ‘fibers’ that could be oriented in eight possible bins (in increments of *π*/8 radians within the range [0, *π*] to reflect the nematic behavior of the system). When the head of a cell moved over a grid point it would update the fibers contained within the grid point. When establishing the model, we considered where fiber deposition would occur. In S2 Fig we show experimentally that fibronectin is produced throughout the cell and initially deposited in the front half of the cell, well ahead of the nucleus. We therefore chose to have cells depositing matix fibers below their heads. Fibers could be generated, deleted or assigned a different directionality, representing the deposition, proteolytic degradation and rearrangement of the matrix, respectively (Fig 2).

#### Fiber deposition

A cell with orientation *θ* deposits fibers according to the deposition rate in the grid point below its head in the bin which *θ* falls in [5]. If *π* ≤ *θ* ≤ 2*π*, fibers are deposited in the bin in which *θ* − *π* falls, since fibers are apolar.

#### Fiber degradation

All bins in the grid point below a cell’s head will be depleted according to the degradation rate.

#### Fiber rearrangement

Fibers from the two neighboring bins will be moved to the bin which *θ* falls in at the specified rearrangement rate. The bins wrap around, so that if *θ* falls in bin 1, rearrangement will happen with its two neighboring bins: bin 2 and bin 8.

#### Choosing fibers for matrix guidance

Our main innovation to the original Vicsek model is in adding the ability of cells to be guided by the matrix fibers below it whilst simultaneously producing and reorganizing these fibers. We hypothesized that the migratory behavior of fibroblasts would be influenced by the emergent matrix organization and that this feedback would influence the matrix topology that ultimately emerges. To investigate directly if fibroblasts were indeed guided by the underlying ECM, we analyzed the migration of fibroblasts plated on matrices with differing degrees of alignment. Specifically, aligning and non-aligning fibroblasts generated respectively aligned and non-aligned matrix over the course of seven days. These original fibroblasts were then removed and new aligning fibroblasts were randomly seeded on both the aligned and non-aligned matrix (S7 Text). We found that the fibroblasts moving on the aligned matrix had significantly increased persistence as compared with the same fibroblast phenotype moving on the non-aligned matrix (Fig 2a, p = 0.0064, two-tailed t-test). This implies that it is not just the presence of matrix but the topology of the matrix that determines cell motility.

Having established experimental justification for matrix guidance we added this mechanism into the model (Fig 2b and 2c). A cell will flock with fibers in the grid point below its head, the bin is determined according to a Gillespie algorithm that takes into account the density of fibers in each bin; therefore, bins with the highest number of fibers have the highest probability of being chosen. The cell will then flock with the left bound of that bin.

#### Sequence of model updates

The order of events in the simulations at each time-step is given as:

1. Compute new cell orientations
2. Compute and update changes to matrix grid points
3. Compute and update new cell positions.

Model implementation and parameter choice is described in the Supporting Text.

### An overview of quantification of matrix metrics

Here we briefly describe how the five metrics are determined. A full description of their derivation is given in S2 Text.

#### Long-range alignment (LRA) and short-range alignment (SRA)

The alignment for an individual fiber is computed as the median angle of deviation between that fiber and all fibers within a pre-defined neighbourhood length; a longer length for LRA and a shorter length for SRA. The alignment of a matrix image at this specified neighbourhood length is given as the mean of the alignment of individual fibers [5].

#### High density matrix (HDM)

Matrix images are thresholded against a pre-defined value. Pixels above this threshold are considered to be fibers, whilst the remaining pixels are background. HDM is then computed as the percentage of fibers in the image.

#### Curvature (Curv)

For each matrix image, by using a Gaussian filter with a large radius, a mask is derived showing the principal matrix fiber structure. The mask lines are then traversed by increments of a pre-defined curvature window. Curvature at that point, is the change in angle between one line segment and the next. The Curv metric is then the average of curvature values for the entire image [48].

#### Fractal dimension (Frac)

For each matrix image, by using a Gaussian filter with a small radius, a mask is derived showing more in detail matrix fiber structure than the curvature masks. We then computed the box counting fractal dimension of the mask. For boxes of a given size, the number of these boxes required to cover the mask is computed. For boxes with side-length of ever-decreasing sizes, the number of boxes required to cover the mask is computed. Box dimension is then defined as the limit of how the number of required boxes to cover the mask scales with box side-length [45, 48].

### Individual migratory persistence and cell-cell guidance alone can generate alignment

**Fig 3:**
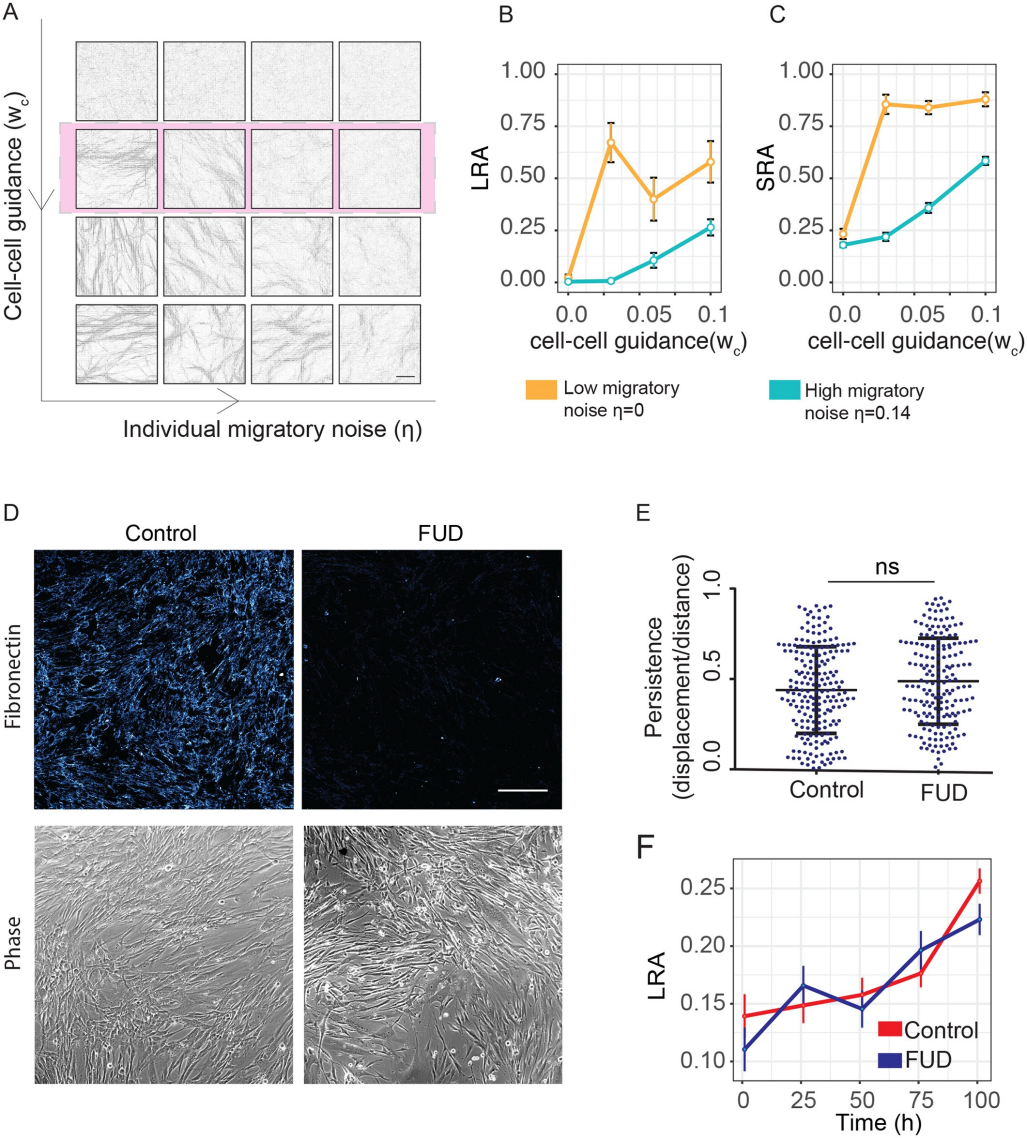
Fibroblasts can align without matrix feedback. (A) Matrix patterns produced from cell-cell interactions alone with varying noise and cell-cell guidance. From left to right noise *η* = (0, 0.07, 0.14, 0.21), from top to bottom *w*_*c*_ = (0, 0.03, 0.06, 0.1). The pink row, showing cell-cell guidance set at *w*_*c*_ = 0.03, is fixed for the rest of the simulations in this manuscript. Scale bar represents 100*μm*. (B) The effect on LRA of increasing cell-cell guidance (*w*_*c*_) for cells with low individual migratory noise (*η* = 0, orange) and high individual migratory noise (*η* = 0.14, blue). N = 5 simulations per point in parameter space. (C) Corresponding effect on SRA or increasing cell-cell guidance. (D) Immunofluorescence of fibronectin deposited by aligning fibroblasts in the presence or absence of a Fibronectin blocking peptide (FUD). FUD blocks ECM formation. Scale bars indicate 500*μ*m. (E) Migratory persistence of aligning cells in the presence or absence of FUD (taken over 16 hour intervals). (F) Long-range alignment over time of fibroblasts in the presence or absence of FUD. Inhibition of ECM formation by FUD treatment did not change rate of alignment.

We initially explored the interplay between cells’ individual migratory noise and cell-cell guidance (Fig 3a, S3 Fig). The fibroblasts still deposited matrix into the second layer of the model, but *w*_*m*_ was set to zero, reflecting the cells moving completely independently of the fibers. The model enables us to see different fibroblast and matrix patterning emerging over time (S1–S4 Videos). The top row of Fig 3a shows that in the absence of cell-cell guidance only isotropic matrix is generated, regardless of the level of individual migratory noise. Increasing the cell-cell guidance term leads to the formation of both anisotropic matrix and a spatially non-uniform distribution of both matrix and cells (Fig 3a, S3 Fig).

Crucially this analysis showed that individual migratory noise (*η*) and cell-cell guidance (*w*_*c*_) alone were not able to generate the wide diversity of patterns seen *in vivo* (Fig 1) [6, 7]. Interestingly, there was surprisingly little variation in curvature amongst patterns and long-range alignment (LRA) and short-range alignment (SRA) are highly correlated (Fig 3b and 3c, S3c Fig). This analysis indicates that noise and cell-cell guidance can generate a limited diversity of patterns, with the main emergent pattern besides isotropic matrix being uniform alignment.

In order to test experimentally if matrix feedback was dispensable for alignment, as the model suggested, we treated fibroblasts that generate an aligned matrix with the ‘functional upstream domain’ (FUD) of *Streptococcus pyogenes*. FUD prevents effective matrix assembly from soluble fibronectin into insoluble fibrils [49]. Cell migration is instructed by this fibril form and not the soluble form. In this way treatment with FUD precludes the cells from being guided by the matrix and we are able to eliminate matrix feedback (Fig 3d). The addition of FUD to fibroblast cultures efficiently prevented matrix fiber bundling but did not alter the migratory persistence of fibroblasts (Fig 3e) or prevent the progressive alignment of fibroblasts over time (Fig 3f). Taken together, these analyses indicate that a limited repertoire of matrix patterns can be generated in the absence of matrix feedback.

### Matrix feedback generates diverse matrix patterns

**Fig 4:**
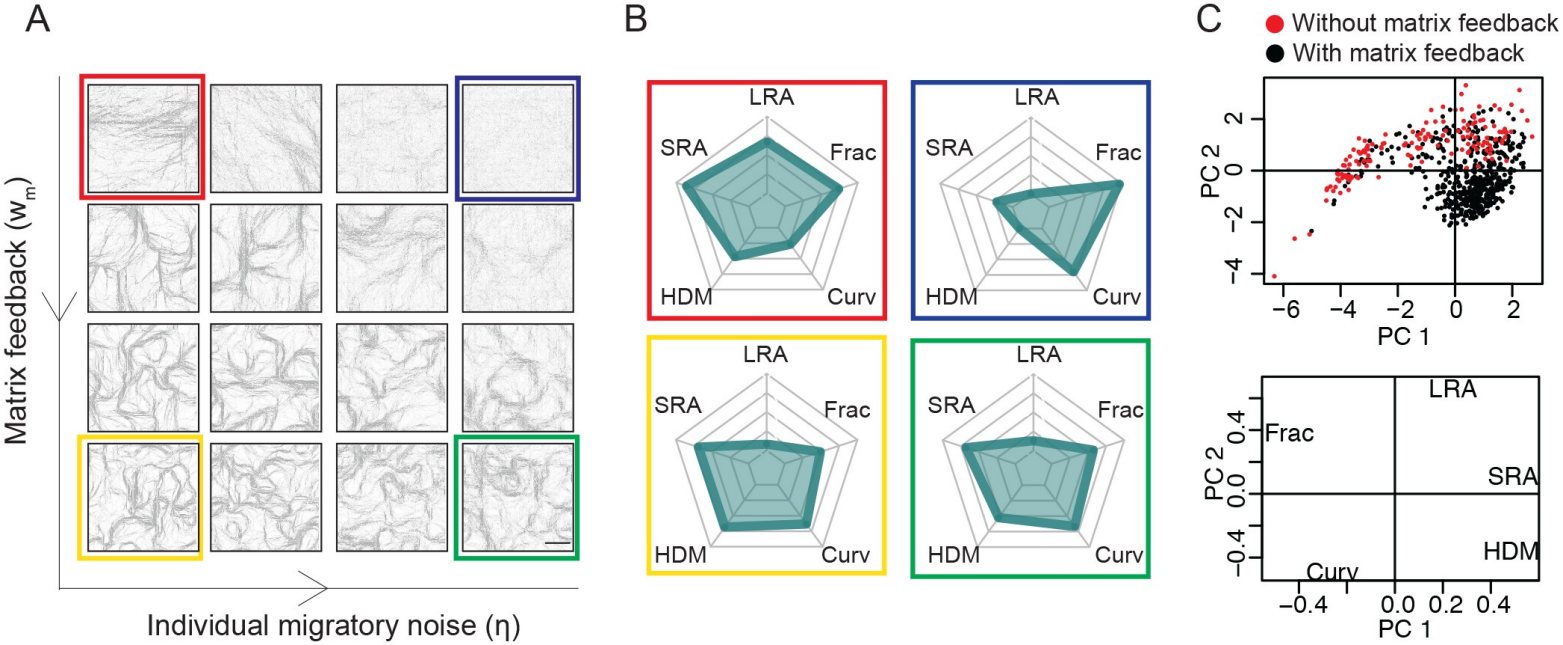
Matrix feedback generates diverse patterning. (A) Matrix patterns produced from varying noise and cell-matrix feedback, cell-cell guidance fixed at *w*_*c*_ = 0.03. Simulations are of 800 cells (40% confluence) over a time-course of seven days. From left to right noise *η* = (0, 0.07, 0.14, 0.21), from top to bottom *w*_*m*_ = (0, 0.04, 0.12, 0.2). Scale bar represents 100*μ*m. (B) Four representative matrix patterns from A are characterized with a starplot according to the metrics of long-range alignment (LRA), short-range alignment (SRA), percentage of high density matrix (HDM), curvature (Curv) and fractal dimension (Frac). (C) PCA of simulations exploring parameter space, reducing metric-space down into two prinicpal components explains 85% of variance and shows the areas in metric-space that can be reached with matrix feedback.

We next considered the effect of introducing varying levels of matrix feedback whilst keeping the level of cell-cell guidance fixed at a level that is consistent with previous work and reflects experimental results [5] (*w*_*c*_ = 0.03 as indicated by the pink strip in Fig 3a). This value of cell-cell guidance provides a physiologically plausible level of coordination between cells such that cells with no individual migratory noise will align and cells with high individual migratory noise will not align. Given the restricted selection of patterns that can be generated by cell-cell guidance alone, we fixed cell-cell guidance at a biologically justifiable level and varied matrix feedback [5]. Fig 4a shows that varying matrix feedback greatly increased the diversity of matrix organization (see also S4 Fig).

Starting with zero matrix feedback, cells with low individual migratory noise produced an aligned matrix characterized by high long-range alignment, high short-range alignment and low curvature (Fig 4a and 4b, red box). Increasing matrix feedback for these cells generated a matrix with high short-range alignment but low long-range alignment, medium curvature and a high proportion of high-density matrix (Fig 4a and 4b, yellow box). As documented in S5 Fig, cells with high individual migratory noise produced a diffuse isotropic matrix with high Frac and Curv and low SRA and LRA, and a low HDM (Fig 4a and 4b, blue box). Increasing matrix feedback for these cells produced a swirl-like but diffuse matrix with high short-range alignment, low long-range alignment and mid-range percentage of high-density matrix and curvature (Fig 4a and 4b, green box).

To investigate further the effect of matrix feedback we performed a comprehensive analysis varying individual migratory noise, cell-cell guidance and matrix feedback together. To obtain an overview of this exploration of parameter space, we used principal component analysis (PCA) to reduce the dimensionality of our matrix metrics [50] (Fig 4c). Dimensionality reduction into two principal components could explain 85% of the variance. The loadings plot shows that the first principal component is related to an increase in short-range alignment, high-density matrix and a decrease in fractal dimension, largely describing the effects of increasing matrix feedback. The second principal component related to an increase in long-range alignment with a small reduction in curvature and high-density matrix, resembling the effects of increasing cell-cell guidance. Importantly, the addition of the matrix feedback term expanded the diversity of matrix outputs visible on the PCA plot (note the expanded region covered by the black dots in Fig 4c). Pairwise analysis of the metrics revealed that the addition of matrix feedback could produce patterns in new areas of metric-space (S5a and S5b Fig). In particular, matrix could now be produced with low long-range alignment but high short-range alignment, high percentage of high-density matrix (30%) and a wide range of curvatures. This describes the spectrum of ‘swirl-like’ patterns created by matrix feedback.

Interestingly, matrix feedback could act as a secondary mechanism for generating short-range alignment of cells with high individual migratory noise (S5b Fig, blue line). However, for cells with low individual migratory noise, matrix feedback actually antagonizes the matrix alignment that would result from the cell-cell guidance term alone. S5b Fig also shows that starting with two cell phenotypes (with high (blue line) and low (yellow line) individual migratory persistence respectively), as matrix feedback increases, the patterning of the two cell-types converges.

Further, an increase in matrix feedback causes the curvature of cells with low migratory noise to increase, fractal dimension of both cell types to decrease and high-density matrix of both cell types to increase (S5b Fig). These changes reflect the increased ‘corralling’ of the cells as they follow and reinforce tracks of matrix fibers that were laid down at early time points (S5 Video). Visually, the result is pronounced swirl-like patterning. The matrix serves as a memory component to the flocking model, causing cells that would eventually normally align through cell-cell guidance to feedback with non-aligned matrix that had been deposited early on, thereby reducing overall alignment.

Similarly, for simulations run at sub-confluence, matrix feedback enhanced diversity of patterns (S5c and S5d Fig). While it is well-documented that cell shape is dynamic through space and time [51, 52], fibroblasts often display an elongated morphology [5, 47]. We hypothesized that different cell shapes in our coarse-grained model would not cause significant perturbations to the system. We confirmed this by running simulations with three distinct cell shapes (S6 Fig) [47]. We excluded the possibility that curvature was caused by the number of grid points comprising the matrix or the number of bins at each grid point by running a subset of simulations with finer grid points and more bins (S2 Text, S7 Fig). In conclusion, these analyses show that matrix feedback increases the diversity of emergent matrix organization, and enables more accurate recapitulation of matrix observed in real tissue.

### How fibroblasts organize the matrix affects pattern formation

We next explored the effect of varying the different components of the matrix organization. In the previous simulations, fibroblasts had just deposited a single fiber at each time step, which had simply increased the number of underlying matrix fibers oriented in the direction of cell migration. We now varied the effect of cell migration on matrix fibers in three different ways: cells could increase the number of fiber elements, cells could re-align existing fiber elements, and cells could degrade existing filament elements, mimicking proteolysis. This manipulation of the second layer of the model allowed us to observe how dynamic remodeling of the ECM might affect matrix patterns by mapping new points in metric space onto the original PCA given in Fig 4c. To reflect the idea that matrix feedback is likely to have a lesser role at early time-points when little ECM has been deposited, we modified the model to take into account matrix feedback as a function of fiber density (see Methods). The original points from Fig 4c are shown faintly on S8a, S8c, S9a and S9c Figs. Analysis was run that combined varying the deposition and rearrangement parameters under conditions of low matrix feedback with either high or low noise (shown in S8 Fig), and high feedback with either high or low noise (shown in S9 Fig).

An increase in deposition rate results in higher HDM and higher curvature, under both high and low feedback regimes. Further, higher deposition rate causes lower LRA as the matrix becomes so dense that there is a loss of order. A higher rearrangement rate leads to fibers being organized into thicker bundles, leading to more high-density matrix especially for cells with high migratory noise (compare yellow and orange boxes S8b and S9b Figs). The effects of matrix reorganization remain largely constant regardless of level of matrix feedback (S8 and S9 Figs). These data demonstrate that varying matrix deposition and re-organization further enhance the diversity of matrix patterns. However, the overall effects of varying how cells reorganize the matrix are subtler than the absolute level of matrix feedback.

### Diverse matrix patterns found *in vivo* can be mimicked *in silico* with matrix feedback

**Fig 5:**
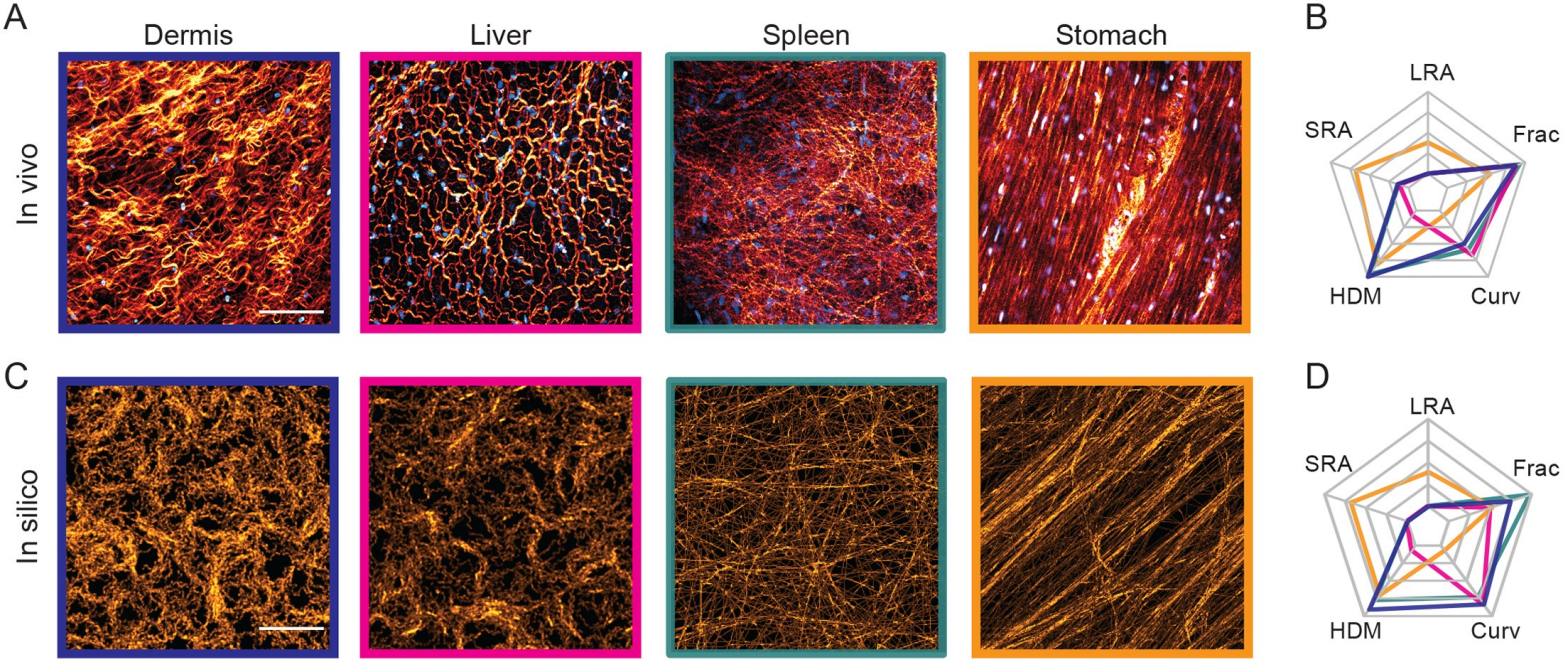
The model can recapitulate ECM diversity *in vivo*. (A) Second harmonic imaging of collagen (orange) in various tissues from a PDGFR nuclear labelled mouse. PDGFR positive nuclei (cyan) indicates fibroblast density. (B) Each matrix is characterized on a starplot below the image according to long-range alignment (LRA), short-range alignment (SRA), percentage of high density matrix (HDM), curvature (Curv) and fractal dimension (Frac). (C) *In silico* matrix that mimics the *in vivo* patterns above. Differences in parameters for generating such matrix patterns are given in Table 1. (D) Corresponding normalized starplot of *in silico* matrix. Scale bars represent 100*μ*m.

We next sought to relate insights from our model to physiologically observed ECM in Fig 1, shown again in Fig 5a and 5b. Through our exploration of parameter space we inferred parameters that would be able to generate similar matrix patterns, using a comparable number of fibroblasts (N = 200 cells, corresponding to 10% confluence) to the *in vivo* systems (Table 1, S5 Text). By reducing cell speed and making the fiber grid twice as fine to increase precision of matrix fiber positioning, the model was able to recapitulate all of these *in vivo* matrix patterns across the same mesoscale (Fig 5c). Nevertheless, given that the *in vivo* matrix patterns are more prone to noise within their biological component features as compared to the more exacting *in silico* simulation studies, great care was taken to pace comparisons on an equal footing, see S6 Text for an in-depth discussion. An interesting discussion on the effects of cell speed on emergent patterning due to distance travelled by cells between samplings can be found in [34].

**Table 1:** Differences in parameter values for generating *in silico* matrix in Fig 5c.

	Dermis	Liver	Spleen	Stomach
Noise (*η*)	0.1	0.1	0.01	0.02
Cell-cell guidance (*w*_*c*_)	0.03	0.03	0	0.03
Matrix feedback (*w*_*m*_)	0.4	0.8	0.01	0

The corralled and spatially heterogeneous patterns observed in the dermis and liver suggest that matrix feedback is required to generate these patterns and their high curvature is indicative of high migratory noise. We have shown that low individual migratory noise and non-zero cell-cell guidance alone are sufficient to generate a highly aligned matrix (Fig 2) like the stomach. The individual strand-like pattern of the spleen suggests a very low level of cell-cell coordination. The starplots characterizing the *in silico* matrix match the *in vivo* starplots closely after normalization (Fig 5d). Importantly, the ‘swirl-like’ patterns seen in the dermis and liver required matrix feedback (Table 1).

### Interconversion between matrix patterns is inhibited by high matrix feedback

**Fig 6:**
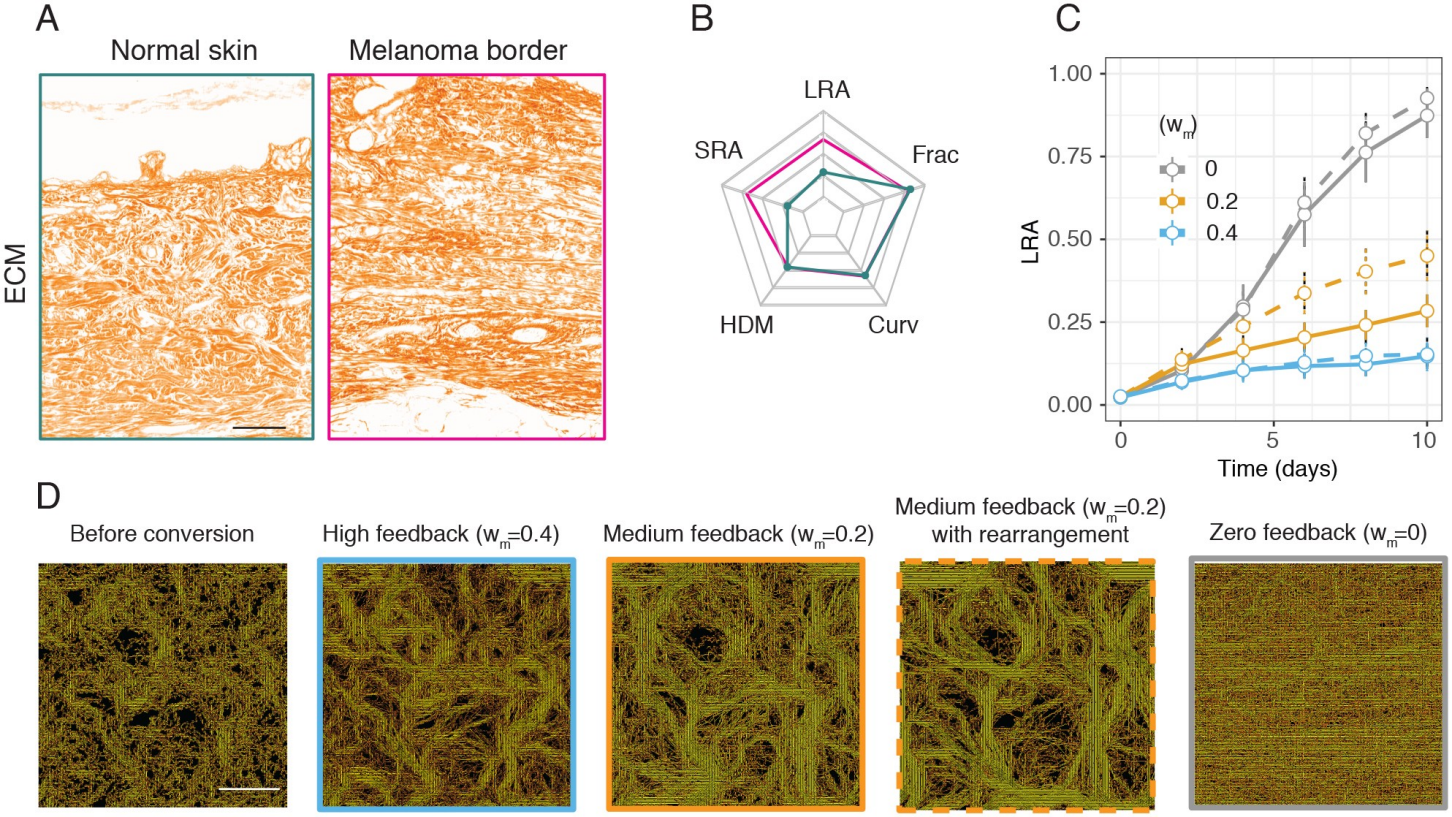
Modelling conversion between matrix patterning *in silico*. (A) Matrix images of normal dermis (left) as compared to the melanoma border (right). (B) Corresponding starplot characterizing normal dermis (green line) and melanoma (pink line). (C) Long-range alignment (LRA) over time beginning with *in silico* dermis for varying levels of matrix feedback: *w*_*m*_ = 0 (grey lines), *w*_*m*_ = 0.2 orange lines, *w*_*m*_ = 0.4 blue lines. Deposition rate = 10. Solid lines show simulations with degradation and rearrangement rates set to zero, whilst dotted lines show simulations with degradation and rearrangement rates set to five. At day 10, when matrix feedback *w*_*m*_ = 0.2, simulations with fiber degradation and rearrangement are significantly more aligned (p-value = 9e-04, two-tailed t-test). N = 25 simulations per point in parameter space. (D) Corresponding *in silico* matrix showing before and after conversion. Colour brightness has been normalized across images. Scale bars correspond to 100*μm*.

Matrix patterns are known to change in cancer and in ageing [7]. Having studied the emergent generation of matrix patterns, we next sought to study the interconversion from one matrix pattern to another. Matrix organization revealed by Gomori trichrome staining of collagen in a normal region of human dermis is characterized by curved matrix fibers, similar to the mouse (Fig 6a). In contrast, a region of dermis from the same individual that is adjacent to a melanoma exhibits altered matrix organization (Fig 6a), with fibers that are more aligned near the melanoma (Fig 6b). Consistent with a previous report that targeted BRAF inhibition in melanoma activates stromal fibroblasts [53], a comparison of ECM organization in the same patient prior to therapy and post-therapy indicated that when the treatment is failing, matrix alignment increases and curvature and fractal dimension decrease (S10 Fig). These analyses show that matrix can be dynamically remodeled over time [6].

Having observed matrix interconversion in pathological samples, we considered it in our model. At high levels, matrix feedback would simply constrain fibroblasts to migrate along existing ECM and preclude changes in matrix pattern. Fig 2 demonstrated that fibroblasts exhibit matrix feedback, but we wanted to determine if this was permissive for matrix remodeling or might be so high that it prevented matrix remodeling. We turned to the model to establish the conditions under which matrix conversion could be achieved. To mimic the situation in melanoma, we explored if an *in silico* generated ‘dermal matrix’ (Fig 5) would be altered by changing the properties of the fibroblasts once the initial dermal matrix had been deposited. As the matrix at the melanoma border exhibited higher alignment (LRA and SRA) than the normal dermis, we reduced the noise of the fibroblasts to zero in the model following the phase of initial matrix deposition. In line with expectation, the model predicts that high levels of matrix feedback will lead to fibroblast migration being channeled into following existing matrix, thereby preventing matrix remodelling (Fig 6c, blue line). However, lowering matrix feedback to 0 permitted a gradual transition from ‘dermal’ matrix to a more aligned matrix (grey line). The emergent matrix showed hybrid features with retention of the original ‘dermal’ matrix (Fig 6c). We therefore explored how adding fiber rearrangement and degradation might interplay with matrix feedback and conversion between matrix patterns (Fig 6c, dotted lines). At a middle level of matrix feedback (*w*_*m*_ = 0.2, Fig 5c orange line), enabling fibroblasts to rearrange and degrade fibers produces a significantly more aligned matrix (p = 9e-04, two tailed t-test). These analyses demonstrate how, when transitioning between two different matrix types, cells can take instruction from the original matrix [34] and produce a hybrid matrix that can have different patterning to *de novo* matrix generation (Fig 6d). In particular, high matrix feedback precludes interconversion by limiting fibroblasts to following existing matrix structures. We therefore conclude that matrix feedback needs to be both non-zero but also not too high. It has the benefit of diversifying matrix pattern, but cannot be so strong as to prevent matrix remodeling.

**Fig 7:**
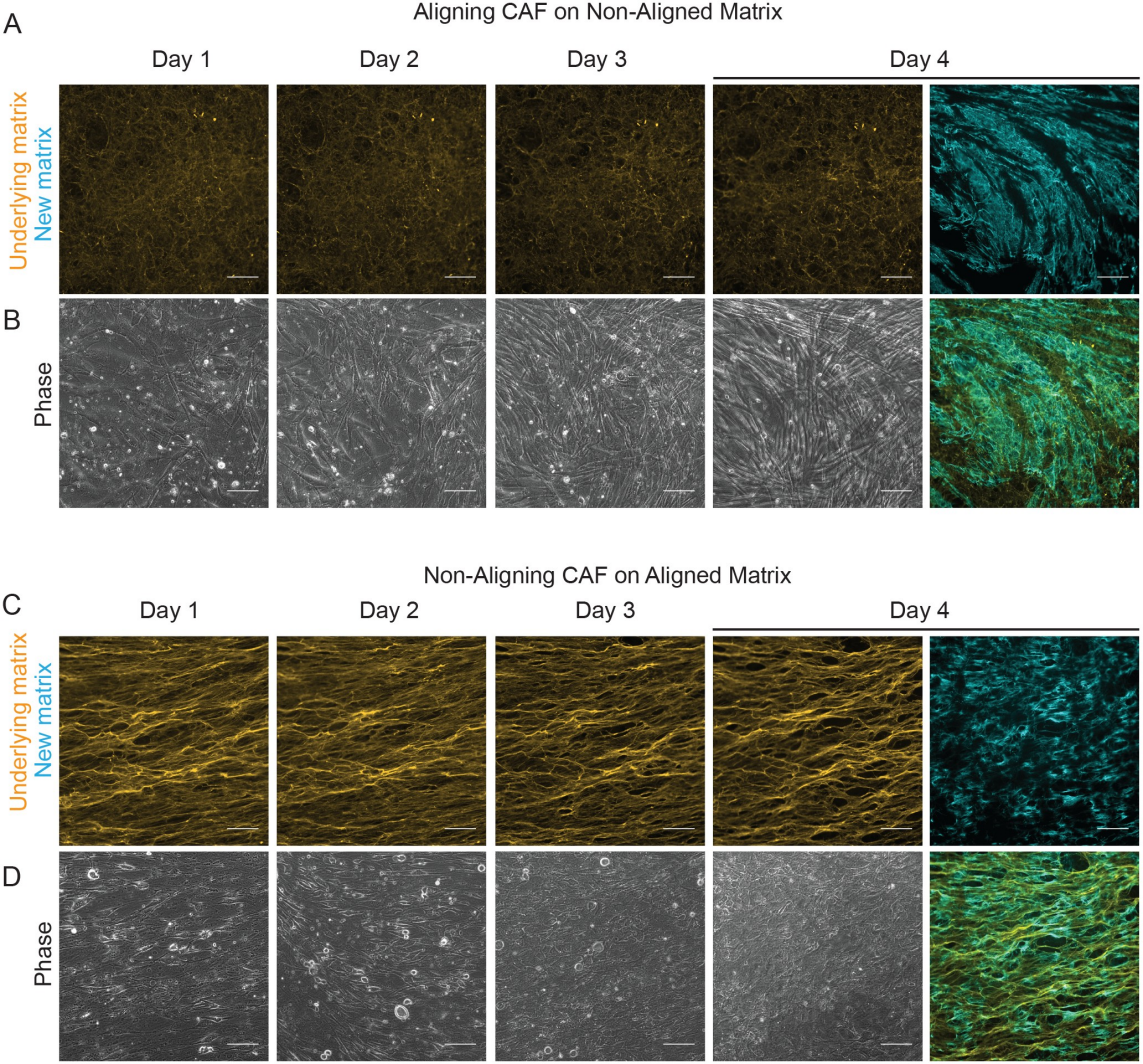
Modelling conversion between matrix patterning *in vitro*. (A) Non-aligning fibroblasts were seeded onto a pre-existing anisotropic matrix. The pre-existing matrix is shown in yellow, new matrix produced over the duration of the assay is shown in blue and the composite image after four days is shown on the right. (B) Corresponding zoomed-in phase imaging of cell-body organization. For the first two days cells follow the matrix and align, but this order breaks down by day 3. (C) Aligning fibroblasts were seeded onto a pre-existing isotropic matrix. (D) Corresponding zoomed-in phase image of cell-body organization. On day 1 cell body orientation is disorganized and follows the isotropic matrix. By day 3 fibroblasts start to ignore the matrix and align. Scale bars represent 100*μm*.

To address this experimentally, we seeded aligning fibroblasts on a thick non-aligned matrix and non-aligning fibroblasts on an aligned matrix, and observed over four days how the cells behaved on the matrix, and the patterning of the new matrix they produced (Fig 7). The original matrix is shown in yellow, with the emergent organization of the fibroblasts at different times shown in phase-contrast below. The new matrix produced by these fibroblasts is shown in blue. In both cases the fibroblasts display an initial tendency to follow the original matrix (day 1), but then revert to their preferred phenotype (day 3-4), being able to ignore the original matrix and produce a new matrix on top. Together, our adapted two-layer nematic Vicsek model and supporting experimental analyses demonstrate that matrix feedback enables diverse emergent patterns, including curved matrix structure, and the strength of matrix feedback is not sufficient to lock the system indefinitely, therefore enabling transitions to occur over a timescale of days.

## Discussion

Simple rules give rise to complex patterns in many natural systems, from Turing’s work on how a zebra gets its stripes to fractal self-similarity in ferns. Previous work [5, 23] has shown how cell-cell interactions can lead to alignment *in vitro*. However, fibroblast and ECM organization will co-evolve *in vivo* in both normal and pathological tissues. Whilst there have been in-depth studies on how fibroblasts organize matrix via contractile forces generating local alignment around a cell [54], or between two contractile cells [5], and the effects of matrix on the movement of individual fibroblasts, surprisingly little is known about how the interplay between these two fundamental mechanisms act in tandem to self-organize. The emergent organization of ECM has critical consequences for tissue function but a mechanistic understanding of how some of these more complex ECM patterns arise is lacking.

To address these issues, we developed a nematic two-layer Vicsek flocking model, explicitly modelling matrix fibers and cells. The extent of matrix feedback and rates of matrix fiber deposition, degradation and rearrangement could all be adjusted in the model. The model enabled us to see both layers, the fibroblasts and the matrix, evolving simultaneously, which remains highly challenging in experimental systems. The model predicts that varying just the cell-cell guidance component without any matrix feedback can generate aligned and isotropic matrix, a finding that we have confirmed experimentally [5]. Introducing matrix feedback increased diversity of tissue patterns, generating matrix with varying long-range alignment, short-range alignment, percentage of high-density matrix, curvature and fractal dimension in metric-space. Matrix guidance of cells occurs irrespective of when the matrix fibers were deposited, resulting in feedback with the matrix acting as temporal ‘memory’ to collective behavior which creates diversity in topology. This temporal dimension of the model together with the ability to read and write between the two layers of fibroblast and matrix are novel extensions to the Vicsek model. Importantly, many of the patterns generated by the model are highly reminiscent of *in vivo* tissues at the mesoscale across millimeters. Recent work on dense suspensions of active filaments has shown it is also possible to produce swirl-like patterns of active filaments for specific values of persistence length and Péclet number [55], however this is dependent on a high packing fraction. Conversely, our model is able to produce diverse patterning even at low confluence, closer to cell density in certain tissues *in vivo*.

It has been hypothesized that matrix feedback could cause fibroblasts to align [22]. Our experiments suggest that it is cell-cell interactions which drive initial alignment without a requirement for ECM, but that matrix feedback plays a crucial role in determining cell motility when the matrix is thick and can facilitate alignment under specific conditions. How matrix feedback affects cell speed is not explored here, however a change in cell speed could affect the cells interaction with matrix, affecting the memory component of matrix feedback. These more nuanced effects of matrix feedback would be a topic of future interest.

Whilst the relatively few rules of the system allows for mathematical tractability and modelling on the scale of millimeters, the model lacks some more subtle matrix features such as matrix cross-linking, which we expect would act to limit matrix reorganization, and environmental cues such as chemoattractants in wound healing [32, 33] and in collective behaviour of dictyoselium [44]. Additional work in this direction could add further insights into the effects of these phenomena. It would be of particular interest to use this model to better understand the transition between matrix topologies as seen in development [10, 11] and ageing. A natural extension of the model would be to move into three dimensions, in order to more clearly understand the *in vivo* context. In three dimensions cell motility is different [56, 57], and we conjecture that matrix feedback would be even stronger due to cells moving through, rather than on top of, matrix. However, continuing to model at the mesoscale level in three-dimensions would be computationally expensive. Finally, it would be interesting to use a deep learning network to see how well the model matches to matrix evolution *in vitro* and *in vivo* and how the model parameters change in these environments. Unlike other modeling approaches which take the ECM to be a surrounding fluid [22, 28–30, 32, 34], our two-layer Vicsek model has met the need for explicit representation of cells and matrix fibers, whilst remaining computationally inexpensive.

By coupling cell-cell and cell-matrix interactions together, we have shown how simple rules can produce diverse tissue patterns. The model offers a potential hypothesis for how many of the complex patterns seen *in vivo* could arise at the mesoscale level. Our multi-layered flocking model unifies cell and matrix behaviors and provides new mechanistic understanding of the consequences of matrix feedback, including its importance for curved matrix patterns and how high levels of feedback hamper matrix remodeling. Such observations are likely to prove important as robust strategies in tissue engineering [2], where the role of ECM-mediated growth and repair is of central importance.

## Methods

### Software and visualization

The model is written in C++. Data analysis was carried out in R and image processing was done in Fiji (ImageJ). Visualization of the matrix is done using openGL. For each grid point, a line showing the orientation of the density of the most recently selected bin is shown, with a color gradation from white to black as a function of that density up to a cut off of 25, which corresponds to a black line. Example visual output is shown in S1 Fig.

### Computing persistence

To avoid bias in computing cell persistence due to cell collisions, cells were tracked at low levels of confluence. The program Metamorph was used for cell tracking. A range of time intervals *T* was chosen over which persistence should be computed. At each point time *t*, the persistence was taken to be the mean directionality ratio of all cells, computed as

$$\begin{array}{c} P\left(t\right)=\frac{|(x_{i}\left(t\right)-x_{i}\left(t-T\right)|}{\sum_{k=1}^{T}|(x_{i}\left(t\right)-x_{i}\left(t-k\right)|} \end{array}$$ (11)

### Varying matrix feedback as a function of fiber density

We computed the persistence of sub-confluent cells moving on glass and on a thick aligned matrix (Fig 2a) for windows of one and two hours. Cells tracked for at least one hour were recorded and a spline tracing the cell’s trajectory was produced using the loess package in R (*α* = 0.5). This was in order to smooth the intracellular movement, which resulted in many small fluctuations in the trajectories. For each cell, the median persistence for each cell was computed as described above. Simulations of single cells with varying values of *Var*(*η*) were run and the persistence computed for one and two-hour windows. The experimental results were then matched with simulations using a least squares approach to select the most likely value of *Var*(*η*) given a cell’s persistence. The distribution of noise in the simulations that matched the cells moving on glass was *Var*(*η*) = 0.13. Simulations were then run for the same level of noise and incremental values of *w*_*m*_ between 0 and 1. The persistence of these simulations was then compared with the persistence of the cells moving on thick aligned matrix. Using a least squares approach, the most likely value of matrix feedback on a thick matrix is *w*_*m*_ = 0.27.

Fig 3e and 3f suggest that for the duration of the four-day FUD experiment (where there is zero matrix guidance) as compared to the control experiment, there was no difference in the persistence or the emergence of alignment. We therefore assume that the control fibroblasts undergo little to no matrix guidance with matrix fibers produced for these first four days. Defining this mathematically we say that for days 1-4, *w*_*m*_ *f*(*d*) = 0, where *d* is fiber density.

On the other hand, as described above, matrix feedback is estimated to be *w*_*m*_ = 0.27 for an aligned matrix produced over seven days. Defining this mathematically we say that by the end of day 7, *w*_*m*_ *f*(*d*) = 0.27, where *d* is fiber density. Using these two way-points at day 4 and day 7, we then ran simulations computing average fiber density across the entire matrix at different times. We took *d* to be the number of fibers in the bin selected for matrix guidance in each matrix grid point. Simulations showed that the average fiber densities could be defined as

$$\begin{array}{c} \bar{d}=\{\begin{array}{cc} 5 & \text{at}\;\text{day}\;\text{4,}\\ 10 & \text{at}\;\text{day}\;\text{7}. \end{array} \end{array}$$

We then used the way-points to define a linear function with conditions *f*(5) = 0 and *f*(10) = 1, where *w*_*m*_ would then be a user-defined maximal level of matrix feedback. We therefore write this function as:

$$\begin{array}{c} f\left(d\right)=\{\begin{array}{cc} 0 & \;\text{if}\;d\leq 5\text{,}\\ \frac{d-5}{5} & \text{if}\;5\leq d\leq 10,\\ 1 & \text{if}\;d\geq 10. \end{array} \end{array}$$

and redefine the X and Y-components of cell i as defined in Eqs (7) and (8), in order that the components will be correctly weighted:

$$\begin{array}{c} X_{i}=\frac{1}{w_{p}+w_{c}+f\left(d\right)w_{m}}\left(w_{p}X_{p}+w_{c}X_{c}+f\left(d\right)w_{m}X_{m}\right), \end{array}$$ (12)

$$\begin{array}{c} Y_{i}=\frac{1}{w_{p}+w_{c}+f\left(d\right)w_{m}}\left(w_{p}Y_{p}+w_{c}Y_{c}+f\left(d\right)w_{m}Y_{m}\right), \end{array}$$ (13)

### Ethics statement

Mice were bred under the authority of Home Office Licence PPL70/8380 granted 2015-2020, which passed CRUK London Research Institute ethical approval in 2014, and culled by a schedule 1 method.

For the melanoma images, tumor samples were collected under the Manchester Cancer Research Centre (MCRC) Biobank ethics application no. 07/H1003/161 + 5 with full informed consent from the patients. The work presented in this manuscript was approved by MCRC Biobank Access Committee application 13-RIMA-01.

## Supporting information

S1 Fig Metrics characterizing matrix. (A) Schematic of patterns with different values of long-range alignment (LRA) and short-range alignment (SRA). (B) Schematic demonstrating different values of high-density matrix (HDM). (C) Schematic showing examples of low and high curvature (Curv). (D) Schematic examples of patterns with different fractal dimension (Frac), including a Hilbert curve as an example of a pattern with a fractal dimension of two. from https://commons.wikimedia.org/wiki/File:Hilbert_curve.svg, licensed under Creative Commons.
(TIFF)

S2 Fig Analyzing where fibroblasts deposit fibronectin. Two examples exploring where fibroblasts produce fibronectin. Fibroblasts were plated, and then began to spread and commence migration. They were then fixed and stained for F-actin (bottom left panels) to reveal their dominant protrusion and hence direction of migration, fibronectin (top left panels) and paxillin to reveal points of substrate attachment (top right panels). A composite image is shown in the bottom right panels. Scale bar represents 10*μm*.
(TIFF)

S3 Fig Emergent *in silico* matrix patterns from cell-cell interactions. (A) Aligned matrix generated with parameters *η* = 0, *w*_*c*_ = 0.03, *w*_*m*_ = 0. (B) Isotropic matrix generated with parameters *η* = 0, *w*_*c*_ = 0, *w*_*m*_ = 0. Images from simulations showing fibroblasts (top) and corresponding matrix (bottom) over six days. Scale bar represents 100*μm*. (C) The effect of increasing cell-cell guidance (*w*_*c*_) on matrix organization for cells with low individual migratory noise (*η* = 0, orange) and high individual migratory noise (*η* = 0.14, blue). N = 5 simulations per point in parameter space.
(TIFF)

S4 Fig Matrix and fibroblast patterns emerging over time with matrix feedback. Images from simulations showing fibroblasts (top) and corresponding matrix (bottom) over six days. (A) Swirl-like matrix generated with parameters set at *η* = 0, *w*_*c*_ = 0.03, *w*_*m*_ = 0.2. (B) Diffuse swirl-like matrix generated by *η* = 0.14, *w*_*c*_ = 0, *w*_*m*_ = 0. For all simulations deposition rate = 1, degradation rate = 0, rearrangement rate = 0. Scale bar represents 100*μm*.
(TIFF)

S5 Fig Emergent *in silico* matrix patterns from matrix feedback. (A) Pair-wise analysis comparing metric-space covered by cells without matrix feedback (red) and with matrix feedback (black) showing the differences between patterns. N = 10 simulations per point in parameter space. Matrix patterns produced from varying noise and cell-matrix feedback, cell-cell guidance fixed at *w*_*c*_ = 0.03. Simulations are of 800 cells over a time-course of seven days. (B) The effect of increasing matrix feedback for cells with low individual migratory noise (*η* = 0, orange) and high individual migratory noise (*η* = 0.14, blue). Error bars show 95% confidence intervals. Simulations run with 800 cells and N = 20 simulations per point in parameter space. (C) PCA of sub-confluent simulations into two components explains 82% of variance. (D) Pairwise analysis comparing cells in sub-confluent conditions without matrix feedback (red) against cells with matrix feedback (black) whilst varying cell-cell flocking and noise. Simulations are of 50 cells over a time-course of seven days.
(TIFF)

S6 Fig Exploring the effect of cell shape on the five metrics. (A) Heatmaps showing long-range alignment (LRA) for simulations with CAFs with an elongated, teardrop and rounded morphology (top, middle and bottom rows respectively). Schematics of these cell shapes are shown on the left. In the first column of heatmaps, matrix feedback is fixed at zero (*w*_*m*_ = 0) whilst noise (*η*) and cell-cell guidance (*w*_*c*_) are varied incrementally. In the second column, *w*_*c*_ = 0 whilst *η* and *w*_*m*_ are varied and in the third column, *η* = 0 whilst *w*_*c*_ and *w*_*m*_ are varied. Comparing the heatmaps row-wise shows that a different cell shape causes little difference in LRA. N = 5 simulations per point in parameter space. Simulations are of 500 cells. Parallel analysis is done for short-range alignment (SRA), high-density matrix (HDM), curvature (Curv) and fractal dimension (Frac) in figures B, C, D and E respectively.
(TIFF)

S7 Fig Parameter sensitivity analysis. (A) The effect of increasing cell aspect ratio on matrix organization for cells with low individual migratory noise (*η* = 0, orange) and high individual migratory noise (*η* = 0.14, blue). N = 5 simulations per point in parameter space. Error bars show 95% confidence intervals. Simulations run with 800 cells. (B) Example stills varying number of matrix grid point and the number of bins per grid point with corresponding starplots below. Scale bar represents 100*μm*.
(TIFF)

S8 Fig Analyzing the effects of varying deposition and rearrangement of fibers on matrix pattern formation with low matrix feedback (*w*_*m*_ = 0.04). (A) PCA for aligning cells with low deposition rate (light orange circle, *η* = 0, depRate = 2, degRate = 1, reRate = 0), aligning cells with high deposition rate (dark orange circle, *η* = 0, depRate = 10, degRate = 1, reRate = 0), non-aligning cells with low deposition rate (light blue circle, *η* = 0.14, depRate = 2, degRate = 1, reRate = 0) and non-aligning cells with high deposition rate (dark blue circle, *η* = 0.14, depRate = 10, degRate = 1, reRate = 0). Blue arrow indicates change in deposition rate for non-aligning cells, yellow indicates change in deposition rate for aligning cells. Background points and loadings are from Fig 3c. (B) Corresponding example stills of the matrix produced by different conditions and their starplots. N = 10 simulations per point in parameter space. (C) PCA for aligning cells with low rearrangement rate (light orange circle, *η* = 0, depRate = 1, degRate = 0, reRate = 0), aligning cells with high rearrangement rate (dark orange circle, *η* = 0, depRate = 1, degRate = 0, reRate = 10), non-aligning cells with low rearrangement rate (light blue circle, *η* = 0.14, depRate = 1, degRate = 0, reRate = 0) and non-aligning cells with high rearrangement rate (dark blue circle, *η* = 0.14, depRate = 1, degRate = 0, reRate = 10). Blue arrow indicates change in rearrangement rate for non-aligning cells. Background points and loadings are from Fig 3c. (D) Corresponding example stills of the matrix produced by different conditions and their corresponding starplots. N = 10 simulations per point in parameter space. Scale bars represent 100*μm*.
(TIFF)

S9 Fig Analyzing the effects of varying deposition and rearrangement of fibers on matrix pattern formation with high matrix feedback (*w*_*m*_ = 0.2). (A) PCA for aligning cells with low deposition rate (light orange circle, *η* = 0, depRate = 2, degRate = 1, reRate = 0), aligning cells with high deposition rate (dark orange circle, *η* = 0, depRate = 10, degRate = 1, reRate = 0), non-aligning cells with low deposition rate (light blue circle, *η* = 0.14, depRate = 2, degRate = 1, reRate = 0) and non-aligning cells with high deposition rate (dark blue circle, *η* = 0.14, depRate = 10, degRate = 1, reRate = 0). Blue arrow indicates change in deposition rate for non-aligning cells, yellow indicates change in deposition rate for aligning cells. Background points and loadings are from Fig 3e. (B) Corresponding example stills of the matrix produced by different conditions and their corresponding starplots. N = 10 simulations per point in parameter space. (C) PCA for aligning cells with low rearrangement rate (light orange circle, *η* = 0, depRate = 1, degRate = 0, reRate = 0), aligning cells with high rearrangement rate (dark orange circle, *η* = 0, depRate = 1, degRate = 0, reRate = 10), non-aligning cells with low rearrangement rate (light blue circle, *η* = 0.14, depRate = 1, degRate = 0, reRate = 0) and non-aligning cells with high rearrangement rate (dark blue circle, *η* = 0.14, depRate = 1, degRate = 0, reRate = 10). Blue arrow indicates change in deposition rate for non-aligning cells. Background points and loadings are from Fig 3e. (D) Corresponding example stills of the matrix produced by different conditions and their corresponding starplots. N = 10 simulations per point in parameter space. Scale bars represent 100*μm*.
(TIFF)

S10 Fig *In vivo* matrix is remodelled in disease and ageing. (A) Zoomed in example simulation image showing matrix in orange and elliptical cell nuclei in blue oriented in the direction of travel of the cell (left) as compared with the *in vivo* image of the murine stomach (right). Scale bars represent 25*μm*. (B) Second harmonic imaging of collagen (orange) in young and old mouse dermis (C) Corresponding starplots. (D) Gomori Trichrome staining showing from left to right: normal dermis, melanoma border, pre-therapy and post-therapy resistance with matrix shown in blue. Epidermis is denoted with an ‘e’, dermis with a ‘d’ and melanoma with an ‘m’. Corresponding staining of the matrix alone is shown in Fig 5a. Scale bars represent 100*μm*.
(TIFF)

S1 Text Simulation setup. (DOCX)

S2 Text Quantification of matrix patterns. (DOCX)

S3 Text Exploring parameter space. (DOCX)

S4 Text Fibroblast organization of the matrix fibers affects pattern formation. (DOCX)

S5 Text Raw values and normalization of matrix metrics. (DOCX)

S6 Text Choosing parameters for mimicking *in vivo* tissues. (DOCX)

S7 Text Experimental methods. (DOCX)

S1 Video Video showing matrix output of simulation of seven-day assay. Parameters are set at *η* = 0, *w*_*c*_ = 0.03, *w*_*m*_ = 0.2 with deposition rate = 1, rearrangement rate = 0, degradation rate = 0.
(MP4)

S2 Video Video showing cell bodies corresponding to the matrix produced in S1 Video. (MP4)

S3 Video Video showing matrix output of simulation of seven-day assay. Parameters are set at *η* = 0.14, *w*_*c*_ = 0.03, *w*_*m*_ = 0.12 with deposition rate = 2, rearrangement rate = 1, degradation rate = 1.
(MP4)

S4 Video Video showing cell bodies corresponding to the matrix produced in S3 Video. (MP4)

S5 Video Video showing how high matrix feedback leads to corralling of five cells *η* = 0, *w*_*c*_ = 0, *w*_*m*_ = 0.4. (MP4)
