# Supplementary figures and images for: Matrix feedback enables diverse higher-order patterning of the extracellular matrix
Source: PLoS Comput Biol. 2019 Oct 28;15(10):e1007251. doi: 10.1371/journal.pcbi.1007251 (PMC6816557; doi:10.1371/journal.pcbi.1007251)

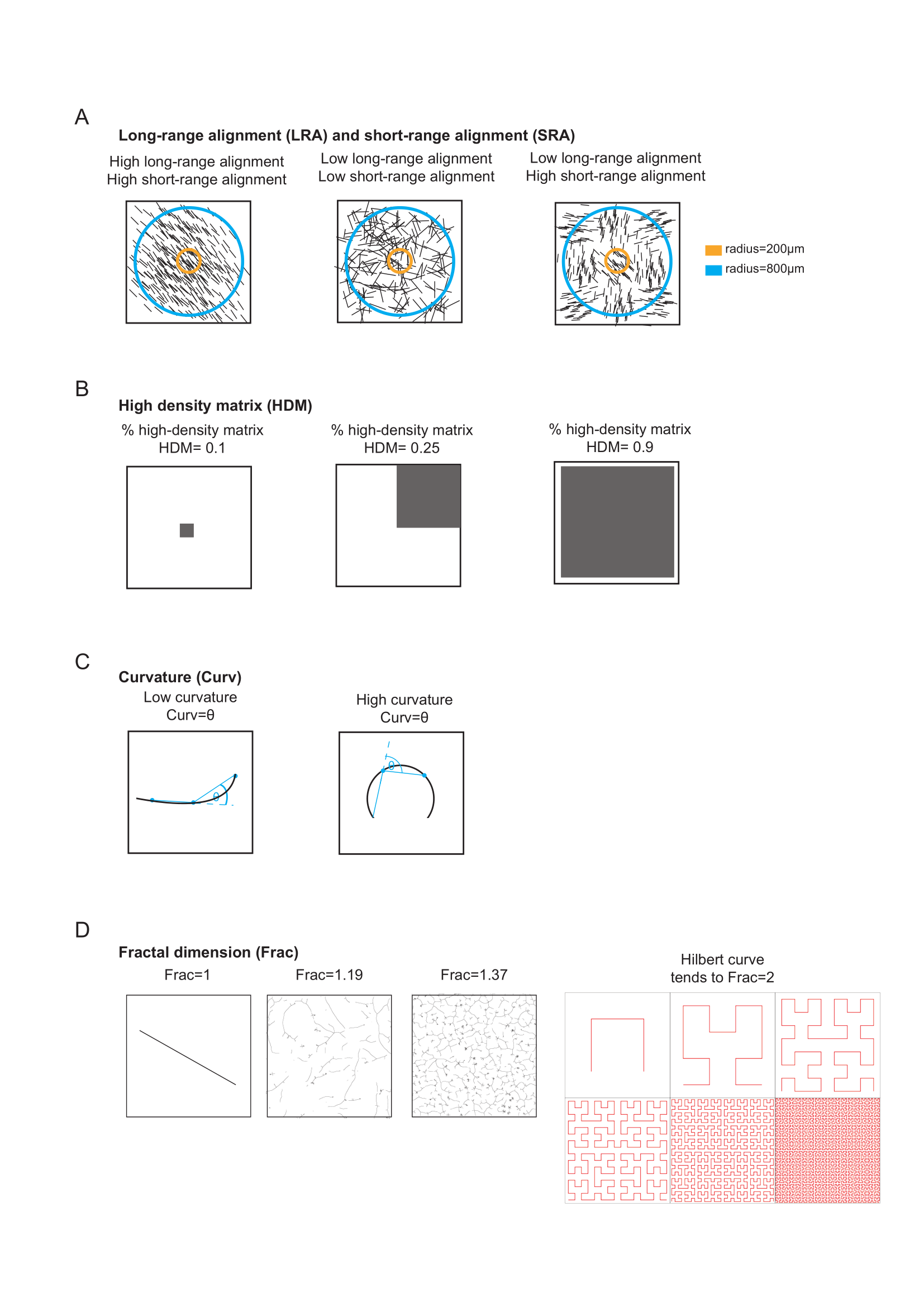

Supplement: S1 Fig — (A) Schematic of patterns with different values of long-range alignment (LRA) and short-range alignment (SRA). (B) Schematic demonstrating different values of high-density matrix (HDM). (C) Schematic showing examples of low and high curvature (Curv). (D) Schematic examples of patterns with different fractal dimension (Frac), including a Hilbert curve as an example of a pattern with a fractal dimension of two. from https://commons.wikimedia.org/wiki/File:Hilbert_curve.svg, licensed under Creative Commons. (TIFF) [file pcbi.1007251.s001.tiff]

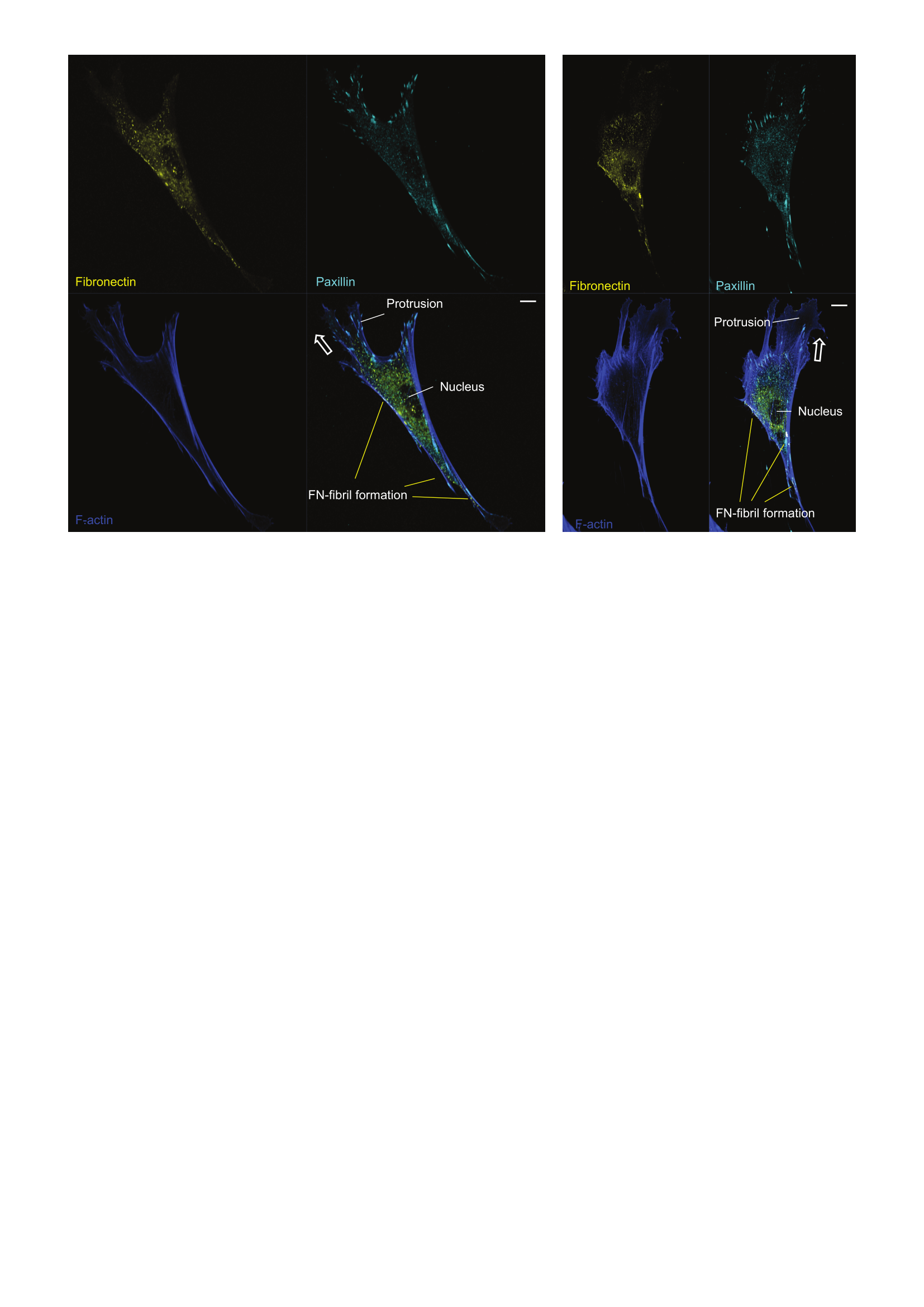

Supplement: S2 Fig — Two examples exploring where fibroblasts produce fibronectin. Fibroblasts were plated, and then began to spread and commence migration. They were then fixed and stained for F-actin (bottom left panels) to reveal their dominant protrusion and hence direction of migration, fibronectin (top left panels) and paxillin to reveal points of substrate attachment (top right panels). A composite image is shown in the bottom right panels. Scale bar represents 10μm. (TIFF) [file pcbi.1007251.s002.tiff]

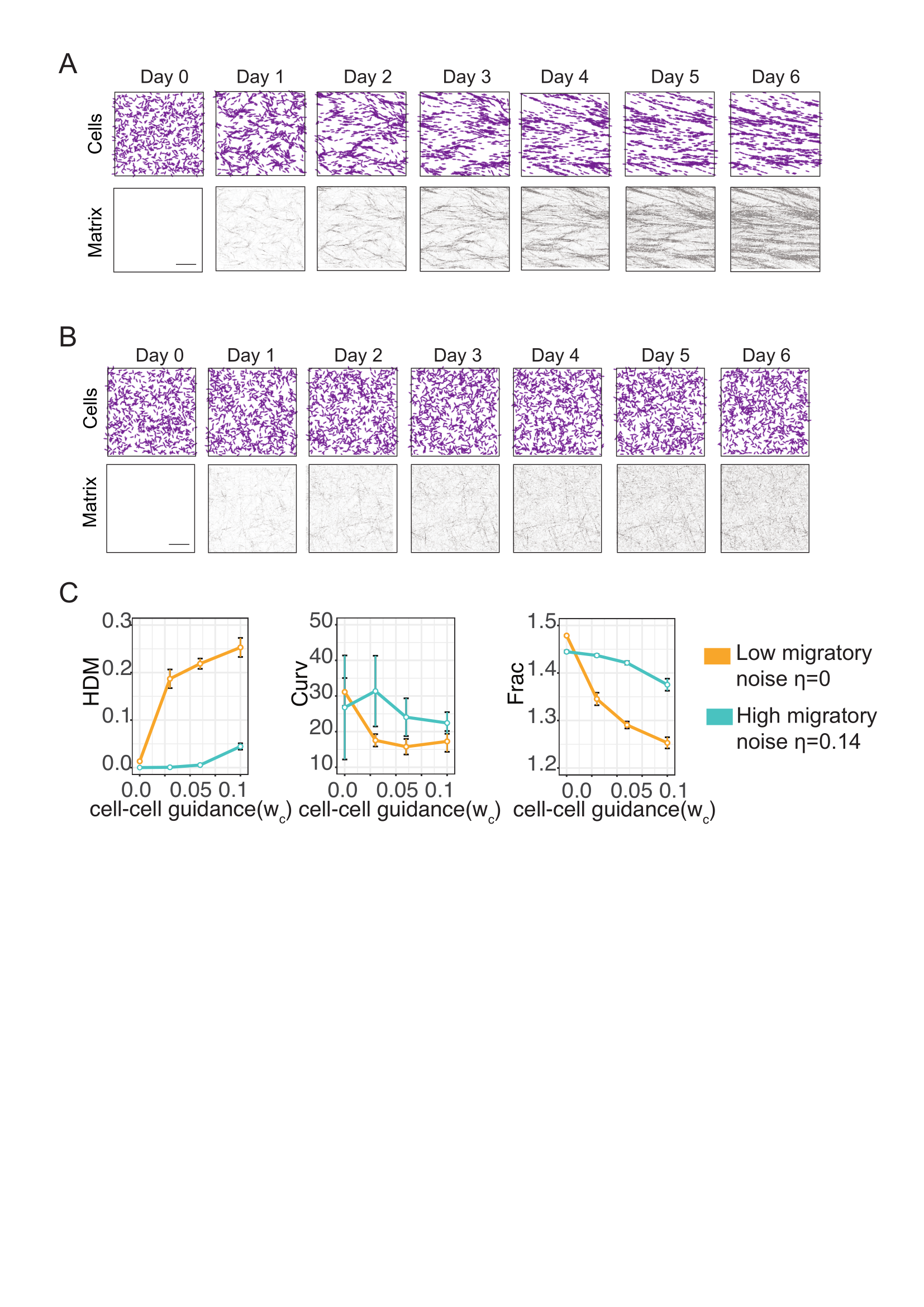

Supplement: S3 Fig — (A) Aligned matrix generated with parameters η = 0, wc = 0.03, wm = 0. (B) Isotropic matrix generated with parameters η = 0, wc = 0, wm = 0. Images from simulations showing fibroblasts (top) and corresponding matrix (bottom) over six days. Scale bar represents 100μm. (C) The effect of increasing cell-cell guidance (wc) on matrix organization for cells with low individual migratory noise (η = 0, orange) and high individual migratory noise (η = 0.14, blue). N = 5 simulations per point in parameter space. (TIFF) [file pcbi.1007251.s003.tiff]

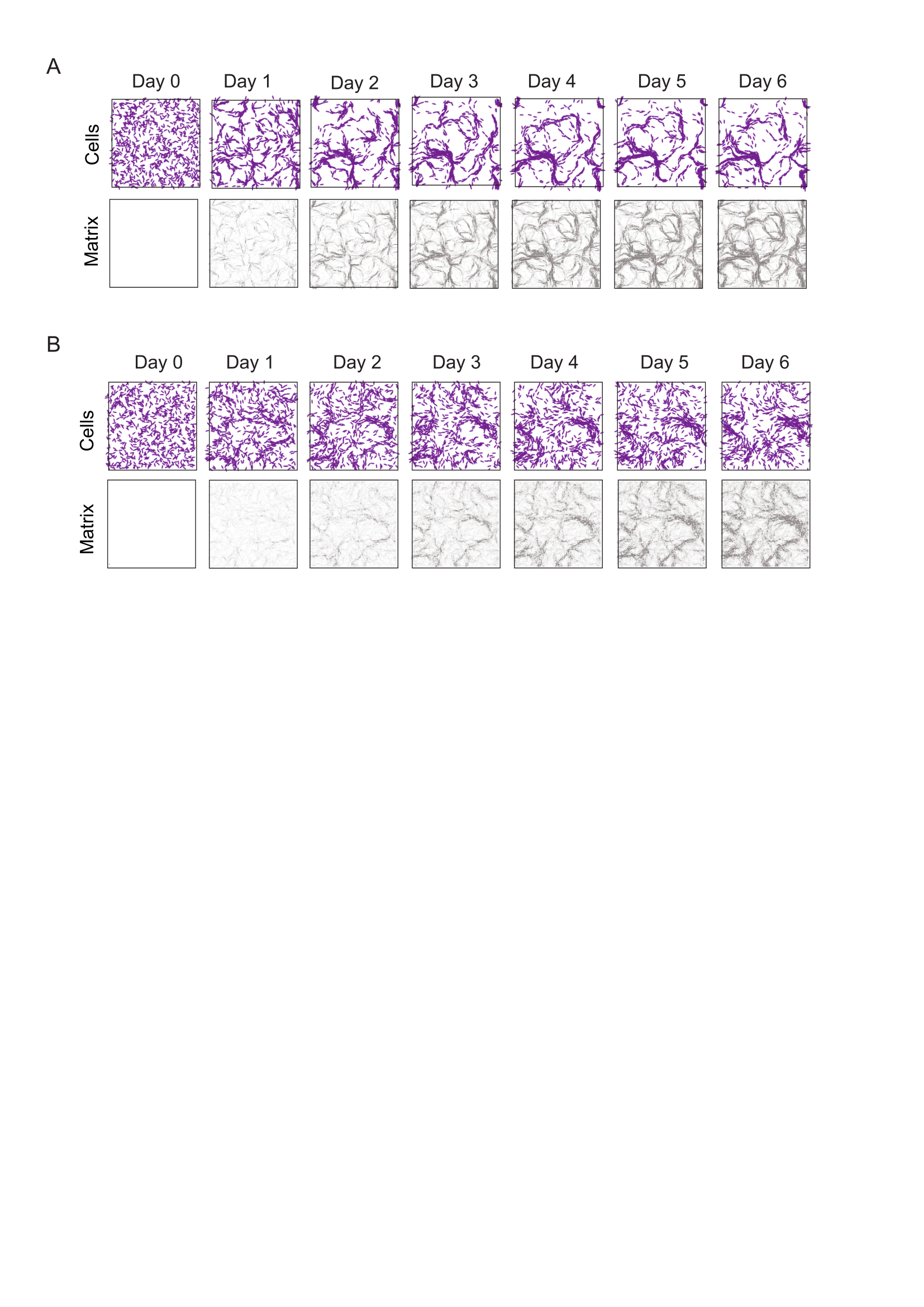

Supplement: S4 Fig — Images from simulations showing fibroblasts (top) and corresponding matrix (bottom) over six days. (A) Swirl-like matrix generated with parameters set at η = 0, wc = 0.03, wm = 0.2. (B) Diffuse swirl-like matrix generated by η = 0.14, wc = 0, wm = 0. For all simulations deposition rate = 1, degradation rate = 0, rearrangement rate = 0. Scale bar represents 100μm. (TIFF) [file pcbi.1007251.s004.tiff]

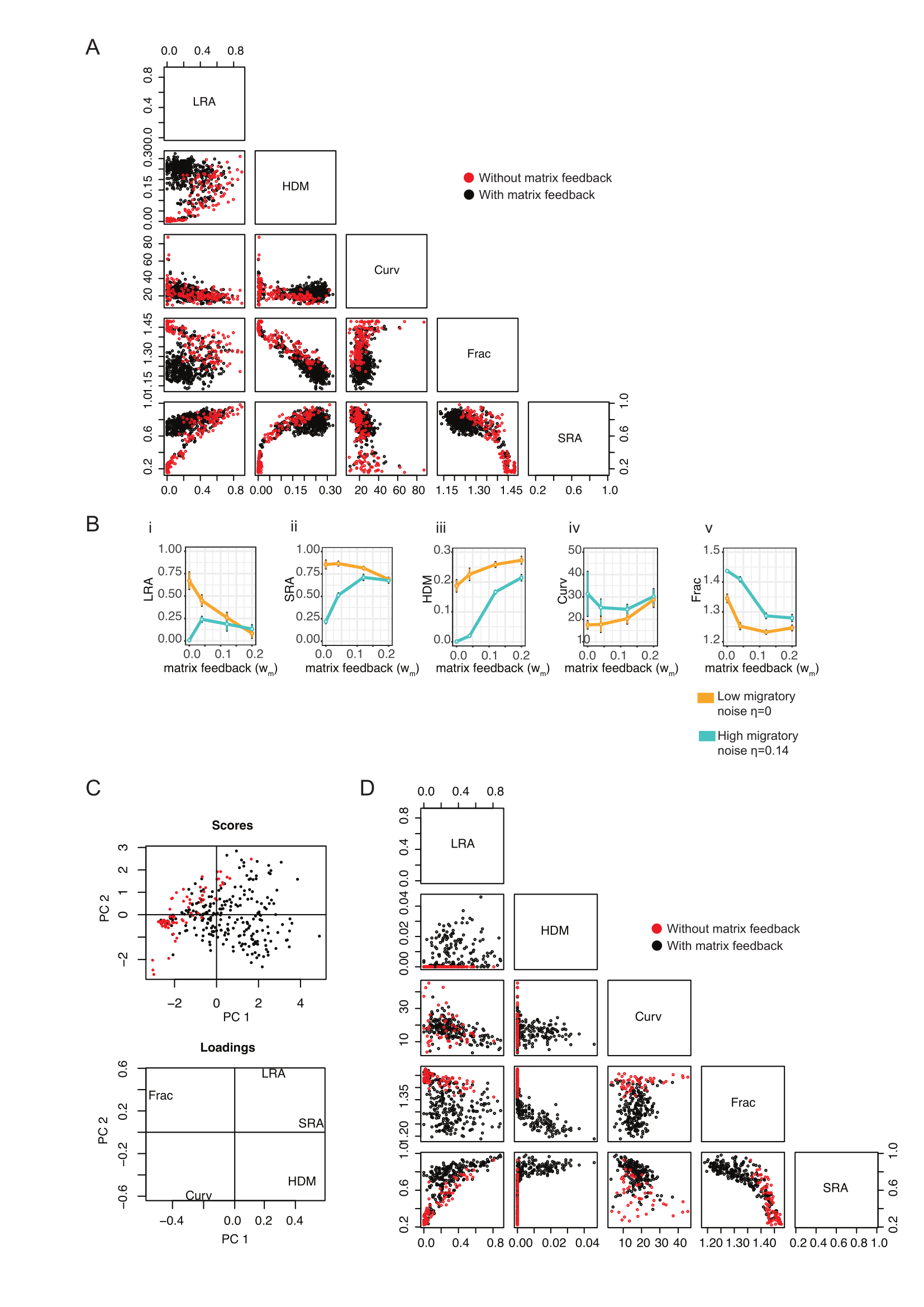

Supplement: S5 Fig — (A) Pair-wise analysis comparing metric-space covered by cells without matrix feedback (red) and with matrix feedback (black) showing the differences between patterns. N = 10 simulations per point in parameter space. Matrix patterns produced from varying noise and cell-matrix feedback, cell-cell guidance fixed at wc = 0.03. Simulations are of 800 cells over a time-course of seven days. (B) The effect of increasing matrix feedback for cells with low individual migratory noise (η = 0, orange) and high individual migratory noise (η = 0.14, blue). Error bars show 95% confidence intervals. Simulations run with 800 cells and N = 20 simulations per point in parameter space. (C) PCA of sub-confluent simulations into two components explains 82% of variance. (D) Pairwise analysis comparing cells in sub-confluent conditions without matrix feedback (red) against cells with matrix feedback (black) whilst varying cell-cell flocking and noise. Simulations are of 50 cells over a time-course of seven days. (TIFF) [file pcbi.1007251.s005.tiff]

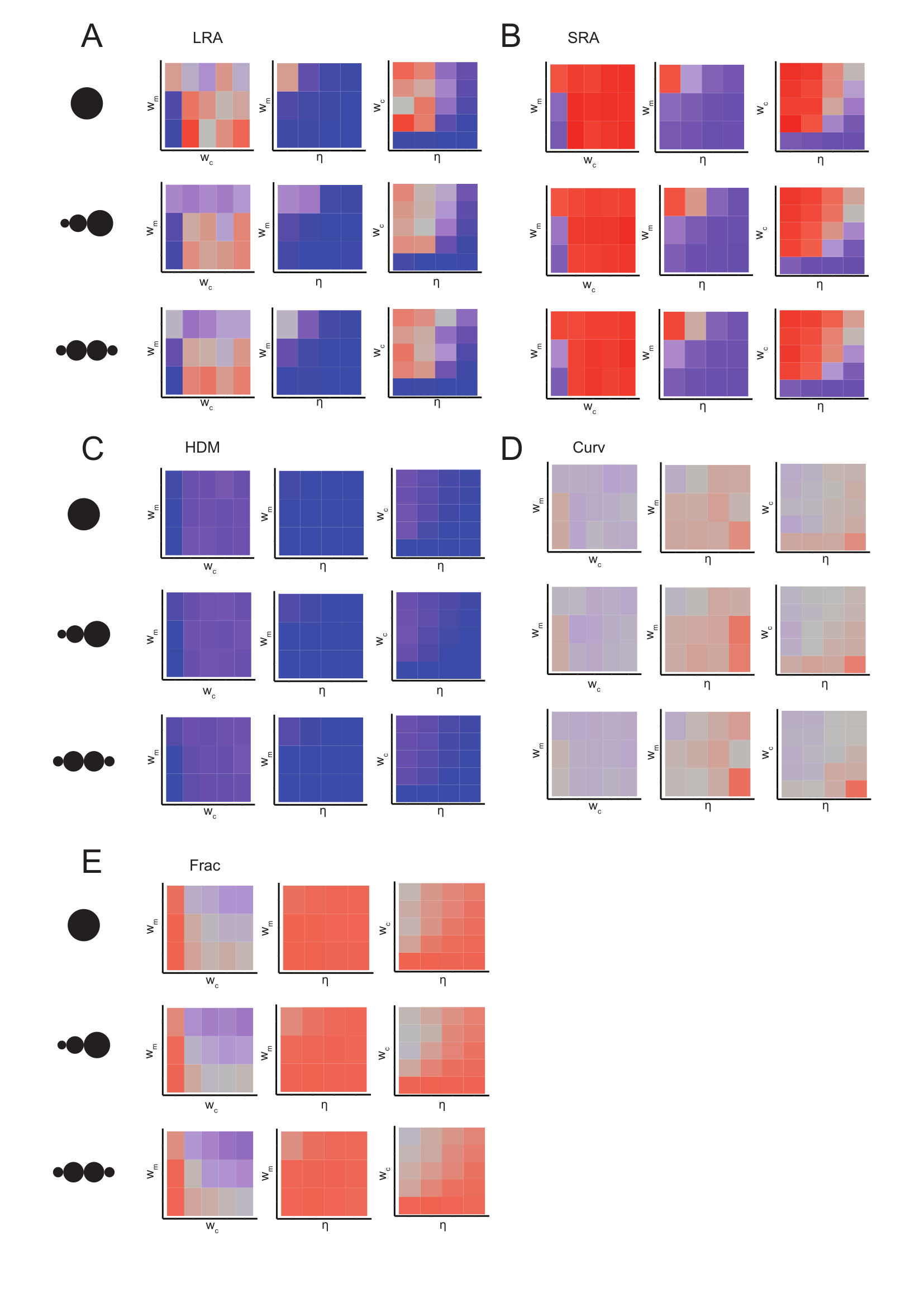

Supplement: S6 Fig — (A) Heatmaps showing long-range alignment (LRA) for simulations with CAFs with an elongated, teardrop and rounded morphology (top, middle and bottom rows respectively). Schematics of these cell shapes are shown on the left. In the first column of heatmaps, matrix feedback is fixed at zero (wm = 0) whilst noise (η) and cell-cell guidance (wc) are varied incrementally. In the second column, wc = 0 whilst η and wm are varied and in the third column, η = 0 whilst wc and wm are varied. Comparing the heatmaps row-wise shows that a different cell shape causes little difference in LRA. N = 5 simulations per point in parameter space. Simulations are of 500 cells. Parallel analysis is done for short-range alignment (SRA), high-density matrix (HDM), curvature (Curv) and fractal dimension (Frac) in figures B, C, D and E respectively. (TIFF) [file pcbi.1007251.s006.tiff]

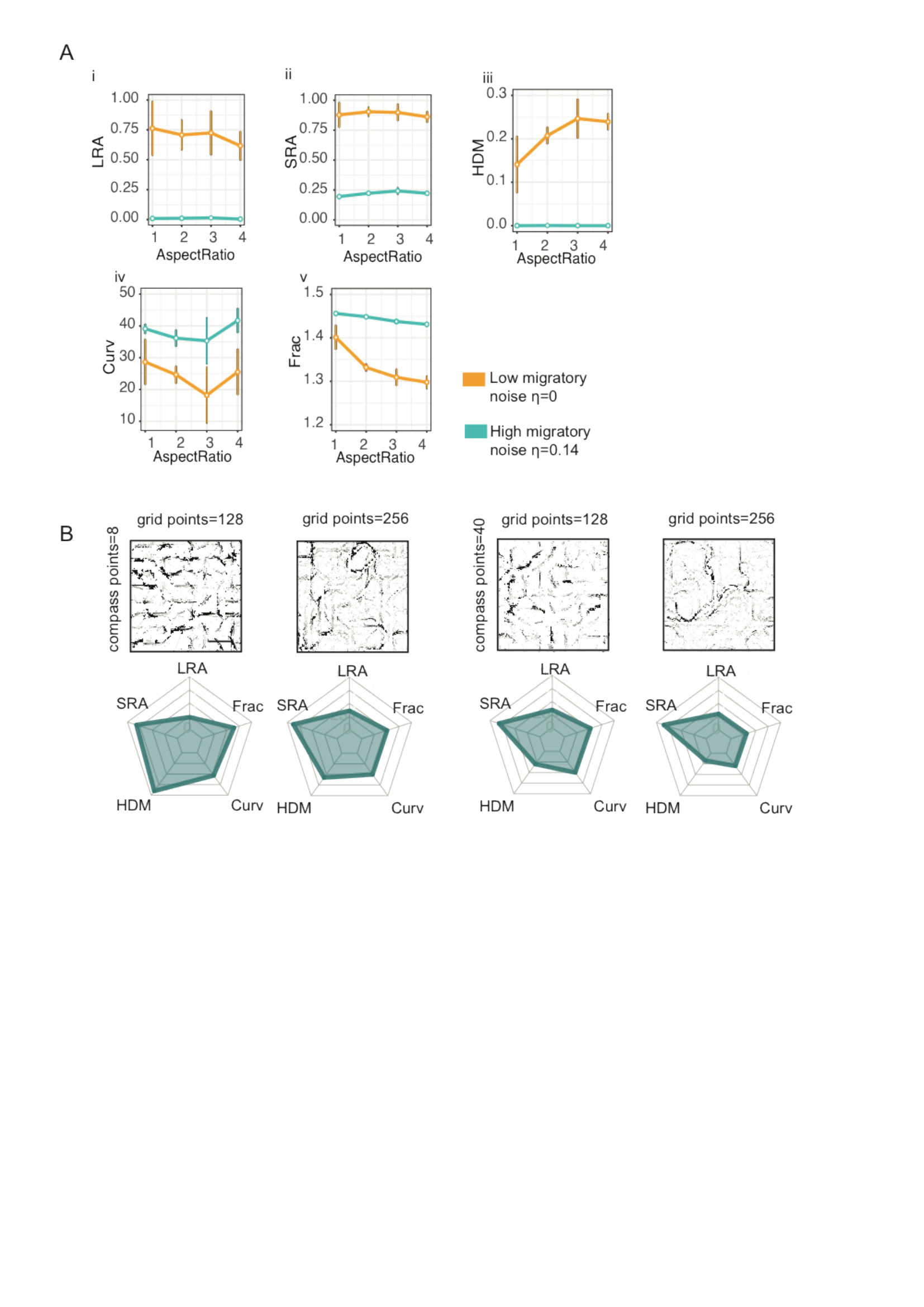

Supplement: S7 Fig — (A) The effect of increasing cell aspect ratio on matrix organization for cells with low individual migratory noise (η = 0, orange) and high individual migratory noise (η = 0.14, blue). N = 5 simulations per point in parameter space. Error bars show 95% confidence intervals. Simulations run with 800 cells. (B) Example stills varying number of matrix grid point and the number of bins per grid point with corresponding starplots below. Scale bar represents 100μm. (TIFF) [file pcbi.1007251.s007.tiff]

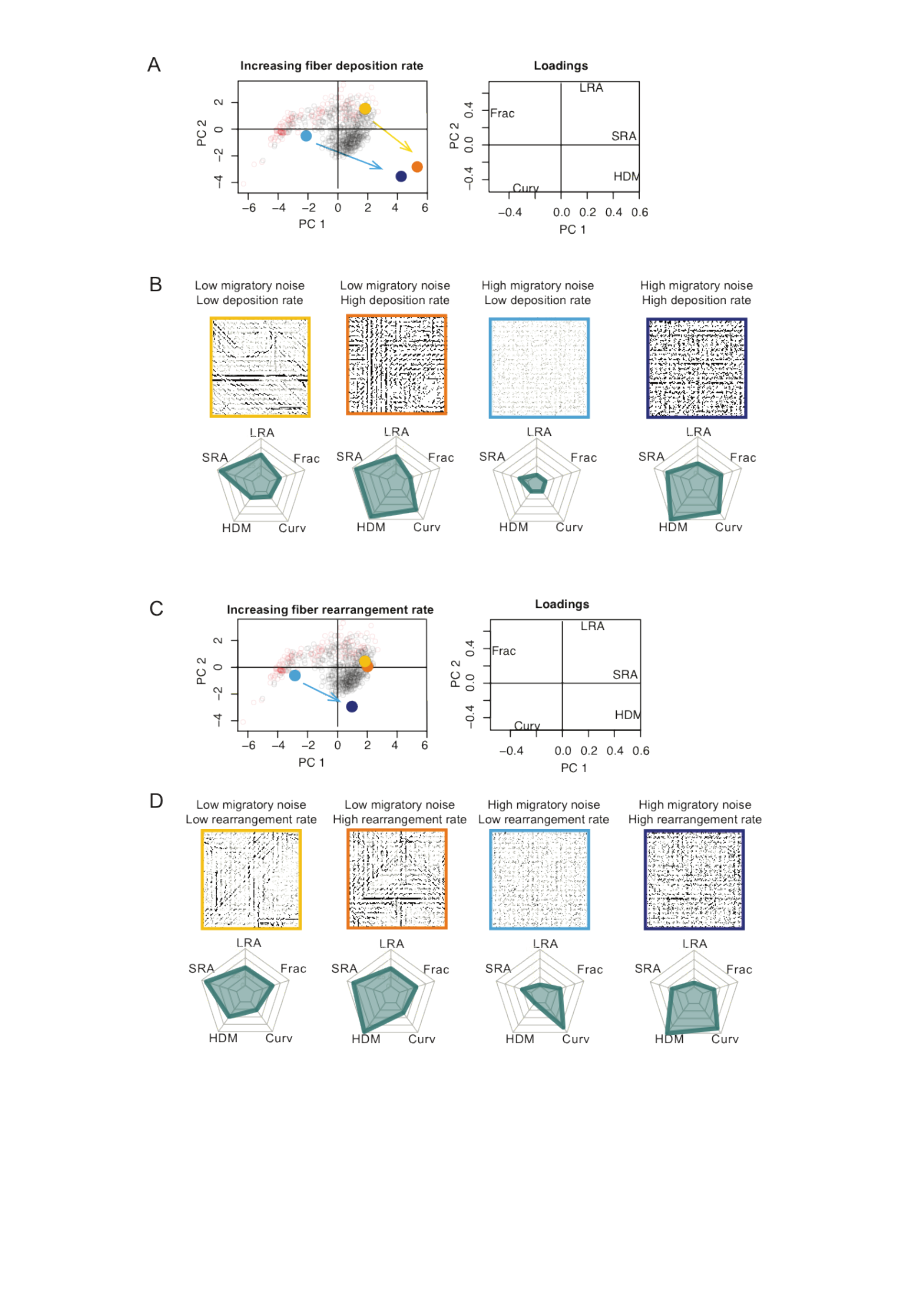

Supplement: S8 Fig — (A) PCA for aligning cells with low deposition rate (light orange circle, η = 0, depRate = 2, degRate = 1, reRate = 0), aligning cells with high deposition rate (dark orange circle, η = 0, depRate = 10, degRate = 1, reRate = 0), non-aligning cells with low deposition rate (light blue circle, η = 0.14, depRate = 2, degRate = 1, reRate = 0) and non-aligning cells with high deposition rate (dark blue circle, η = 0.14, depRate = 10, degRate = 1, reRate = 0). Blue arrow indicates change in deposition rate for non-aligning cells, yellow indicates change in deposition rate for aligning cells. Background points and loadings are from Fig 3c. (B) Corresponding example stills of the matrix produced by different conditions and their starplots. N = 10 simulations per point in parameter space. (C) PCA for aligning cells with low rearrangement rate (light orange circle, η = 0, depRate = 1, degRate = 0, reRate = 0), aligning cells with high rearrangement rate (dark orange circle, η = 0, depRate = 1, degRate = 0, reRate = 10), non-aligning cells with low rearrangement rate (light blue circle, η = 0.14, depRate = 1, degRate = 0, reRate = 0) and non-aligning cells with high rearrangement rate (dark blue circle, η = 0.14, depRate = 1, degRate = 0, reRate = 10). Blue arrow indicates change in rearrangement rate for non-aligning cells. Background points and loadings are from Fig 3c. (D) Corresponding example stills of the matrix produced by different conditions and their corresponding starplots. N = 10 simulations per point in parameter space. Scale bars represent 100μm. (TIFF) [file pcbi.1007251.s008.tiff]

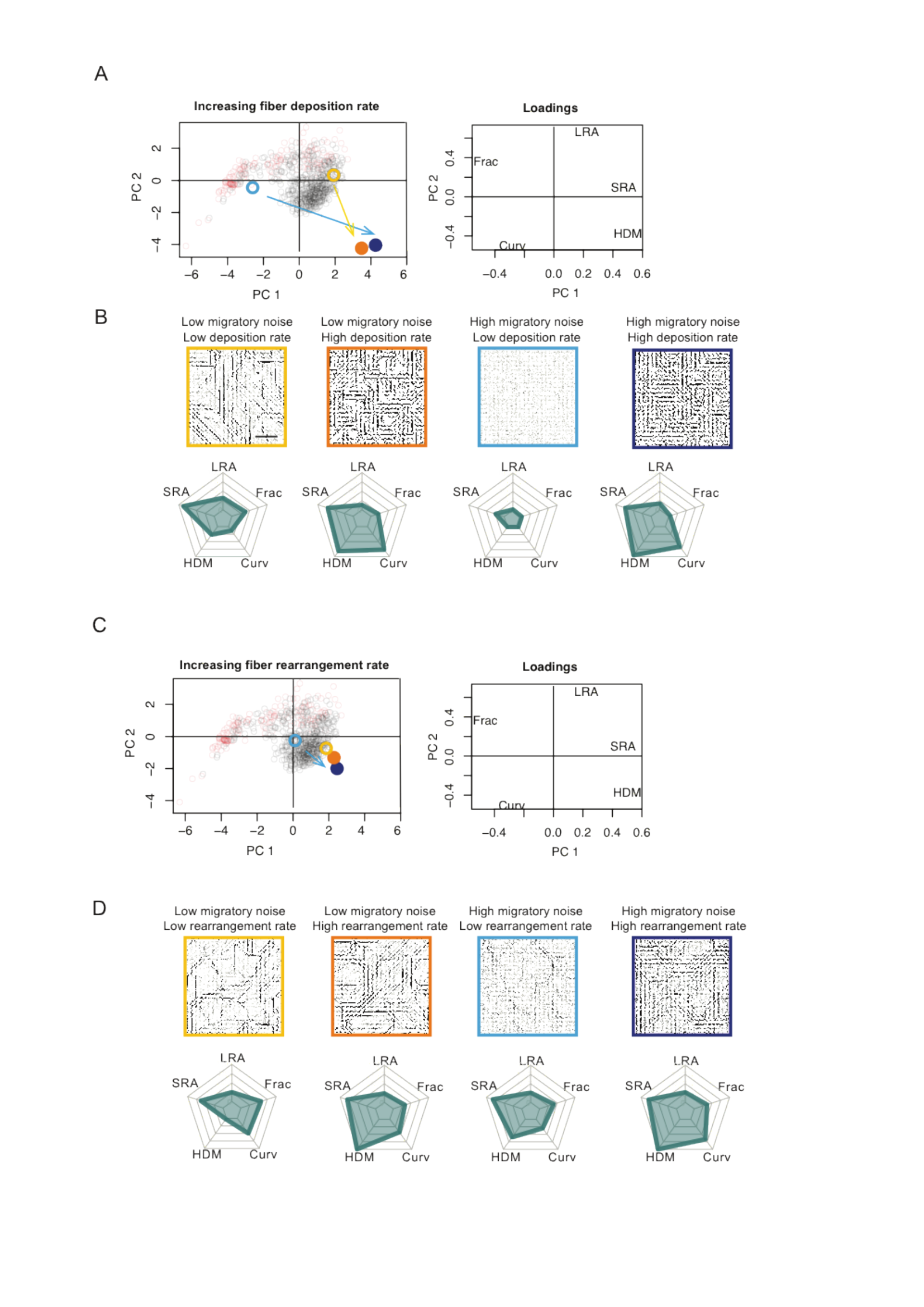

Supplement: S9 Fig — (A) PCA for aligning cells with low deposition rate (light orange circle, η = 0, depRate = 2, degRate = 1, reRate = 0), aligning cells with high deposition rate (dark orange circle, η = 0, depRate = 10, degRate = 1, reRate = 0), non-aligning cells with low deposition rate (light blue circle, η = 0.14, depRate = 2, degRate = 1, reRate = 0) and non-aligning cells with high deposition rate (dark blue circle, η = 0.14, depRate = 10, degRate = 1, reRate = 0). Blue arrow indicates change in deposition rate for non-aligning cells, yellow indicates change in deposition rate for aligning cells. Background points and loadings are from Fig 3e. (B) Corresponding example stills of the matrix produced by different conditions and their corresponding starplots. N = 10 simulations per point in parameter space. (C) PCA for aligning cells with low rearrangement rate (light orange circle, η = 0, depRate = 1, degRate = 0, reRate = 0), aligning cells with high rearrangement rate (dark orange circle, η = 0, depRate = 1, degRate = 0, reRate = 10), non-aligning cells with low rearrangement rate (light blue circle, η = 0.14, depRate = 1, degRate = 0, reRate = 0) and non-aligning cells with high rearrangement rate (dark blue circle, η = 0.14, depRate = 1, degRate = 0, reRate = 10). Blue arrow indicates change in deposition rate for non-aligning cells. Background points and loadings are from Fig 3e. (D) Corresponding example stills of the matrix produced by different conditions and their corresponding starplots. N = 10 simulations per point in parameter space. Scale bars represent 100μm. (TIFF) [file pcbi.1007251.s009.tiff]

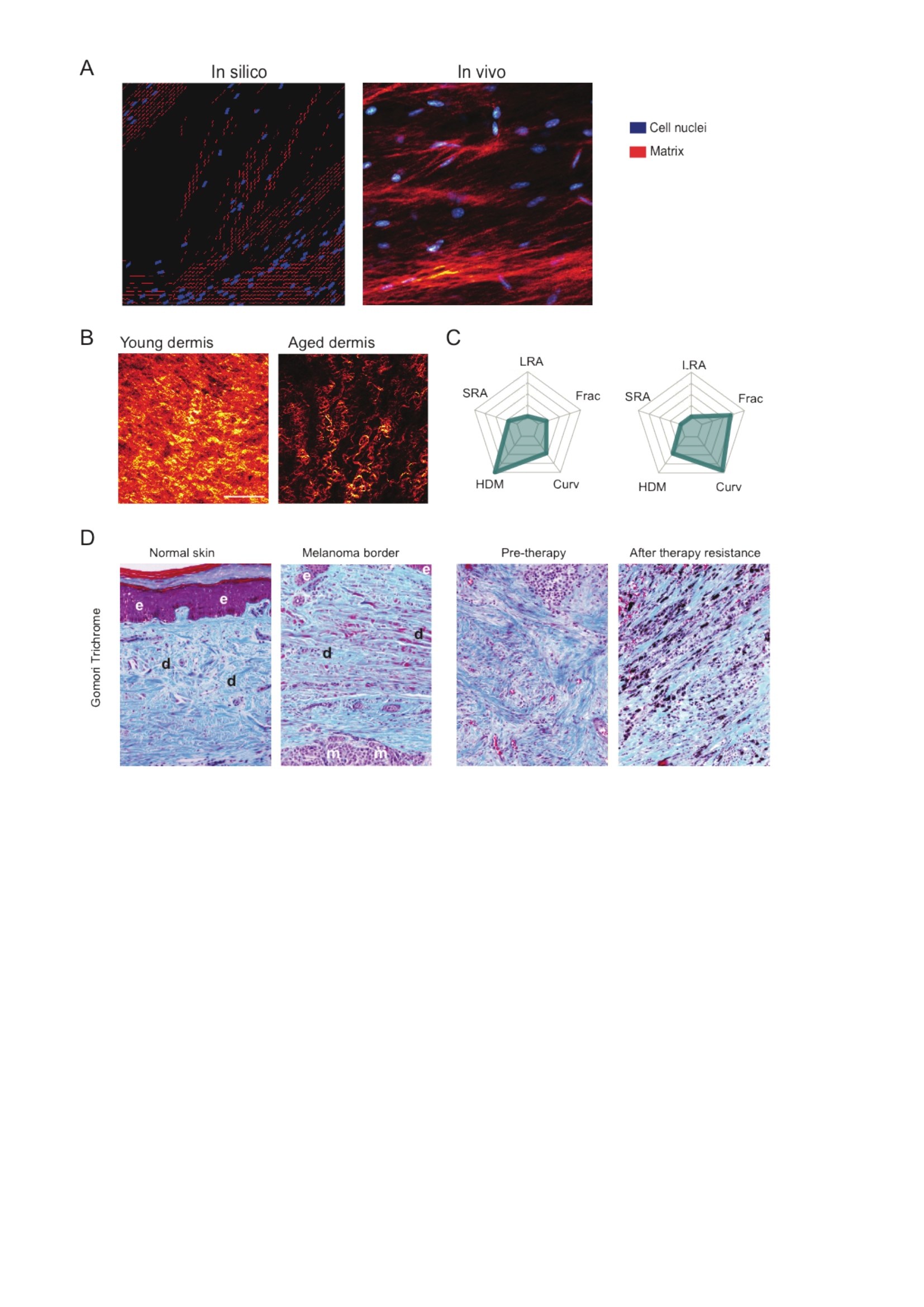

Supplement: S10 Fig — (A) Zoomed in example simulation image showing matrix in orange and elliptical cell nuclei in blue oriented in the direction of travel of the cell (left) as compared with the in vivo image of the murine stomach (right). Scale bars represent 25μm. (B) Second harmonic imaging of collagen (orange) in young and old mouse dermis (C) Corresponding starplots. (D) Gomori Trichrome staining showing from left to right: normal dermis, melanoma border, pre-therapy and post-therapy resistance with matrix shown in blue. Epidermis is denoted with an ‘e’, dermis with a ‘d’ and melanoma with an ‘m’. Corresponding staining of the matrix alone is shown in Fig 5a. Scale bars represent 100μm. (TIFF) [file pcbi.1007251.s010.tiff]
