# Supplementary material for: Matrix feedback enables diverse higher-order patterning of the extracellular matrix
Source: PLoS Comput Biol. 2019 Oct 28;15(10):e1007251. doi: 10.1371/journal.pcbi.1007251 (PMC6816557; doi:10.1371/journal.pcbi.1007251)
Supplement: S1 Text — (DOCX) [file pcbi.1007251.s011.docx]

**Text S1 Simulation Setup**

The simulation window size is $1720\times1720\mu m$ and periodic boundary conditions are employed. Time is modelled discretely such that each time step represents one frame in the experiments, which corresponds to 15 minutes and the model typically simulates 7 days assays with 800 cells, corresponding to 40% confluence. At time $t=0$, cells are seeded randomly with random orientation. The grid points describing the matrix are initialized.

CAFs are represented as two large circles with two smaller circles of half the radius at either end. For most simulations, the total cell area is $1410\mu m$, corresponding to a large circle radius of $13.5\mu m$ and the length of the cell is therefore $80\mu m$.

The coarseness of the overall matrix grid is determined by the number of grid points from which it is constructed. By default, the number of grid points is computed as

$$\#grid points=\left( \frac{simulation box length}{diameter of cell head} \right)^{2}$$

which equates to a total of $\left( \frac{1024}{8} \right)^{2}=16384$ squares.

The number bins per grid point that fibers can be deposited in, can be adjusted. In this work, the number of bins was set to 8. Bins are set at increments of $\pi/8$ radians to reflect the antiparallel behavior of the system. The matrix images produced *in silico* show the density of the most recently chosen bin at each grid point.
