# Supplementary material for: Matrix feedback enables diverse higher-order patterning of the extracellular matrix
Source: PLoS Comput Biol. 2019 Oct 28;15(10):e1007251. doi: 10.1371/journal.pcbi.1007251 (PMC6816557; doi:10.1371/journal.pcbi.1007251)
Supplement: S2 Text — (DOCX) [file pcbi.1007251.s012.docx]

**Text S2 Quantification of matrix patterns**

Visual depictions of the five metrics used to characterize each matrix are given in Fig S2.

***Quantifying Alignment in silico***

A metric was developed to quantify extent of alignment over multiple distances. At the end of each simulation, the 800 grid points with the highest fiber density were recorded. In the case of simulations where there 128x128 grid points, this corresponds to taking the densest 5% of grid points. . In the case of simulations where there 256x256 grid points, this corresponds to taking the densest 1% of grid points. For each grid point $i$, for a given neighborhood $r$ between $r_{min}$ and $r_{max}$, let the set $J$ denote all grid points $j\neq i$ which have a distance in the range $[r_{min},r_{max}]$ from $i$. If the vector $\Delta\Theta_{i}$ denoting the antiparallel difference in angle between the dominant density of grid point $i$ and the grid points in $J$ has length $n>5$, then the angle un-normalised alignment of the system at time $t$ over a given distance $r={[r}_{min},r_{max}]$ can be defined as

|  | $u_{r}\left( t \right)=\frac{1}{N}\sum_{i=1}^{N} median(\Delta\Theta_{i})$ | (16) |
| --- | --- | --- |

This can be normalized to give values between 0 and 1 as

|  | $a_{r}\left( t \right)=min\left( 0,1-\frac{u_{r\left( t \right)}}{\frac{\pi}{4}} \right).$ | (17) |
| --- | --- | --- |
|  |  |  |

For each grid point, the median angle of deviation is used as opposed to the mean in order to be less sensitive to outliers. Measurements for each grid point are only considered valid if the neighborhood contains at least five neighbors (including itself) i.e. alignment in low confluence, is not meaningful.

For the manuscript, the short-range alignment (SRA) and long-range alignment (LRA) neighborhoods are defined as grid points within a distance of $0-200\mu m$ and $200-800\mu m$ respectively. Cells in the simulations have length $80\mu m$. The choice of distance defining SRA corresponds to a distance of approximately one cell length and the choice of distance defining LRA corresponds to a distance between cells of approximately 1-5 cell lengths.

***Quantification of experimental matrix alignment***

CT-FIRE^52^ was used to extract individual fibers from 512x512 greyscale images. These were then fed into a MATLAB script, which computed the orientation of each fiber assuming that it was straight between its start and end point. These angles were then input into a 512x512 mathematical matrix. Interpolation was applied to fill in empty matrix values and followed by a smoothing mean filter. We then computed the median difference in angle for increasing radii. In this manuscript, values at distances of 100$\mu m$ and 200$\mu$m are used. The final output for a given radius was the sine of the median angle difference. At all steps, care was taken to respect the periodicity between angles.

***Quantifying Percentage of high density matrix***

Simulation stills from time $t=7$ days were processed in Fiji (ImageJ) by splitting images into RGB channels and saving them as binary images. We then applied a Gaussian blur $(\sigma=5)$ to smooth the image. Then we adjusted images through thresholding, assuming grey matrix on a white background and adjusting the maximum intensity from 255 to 220. Percentage of high density matrix (HDM) was then computed as the proportion of non-white pixels in the whole image.

***Quantifying Curvature***

For quantification of curvature of the matrix, we took snapshots of the matrix at the end of simulations. We then split images into RGB channels and worked with the red channel images. We converted the images to 32-bit images, applied a Gaussian filter $(\sigma=10)$, reset the colors on the Brightness and Contrast function and converted the image back to an 8-bit image. We then created a mask of the image using the ridge detection plugin with a line width of 30. We used the plugin Anamorf^53^ on these masks, taking a curvature window of 50 pixels, corresponding to $84\mu m$. Curvature is measured as the mean change in angle moving incrementally along individual lines along the mask by a window of 50 pixels. There were instances in parameter space where the matrix was so light and diffuse, for example when individual migratory noise was high and deposition rate and matrix feedback was low that the mask did not pick up any individual lines. In these instances, it was not possible to compute curvature or fractal dimension. In Supplementary Figs 6 and 7, curvature and fractal dimension are drawn as being zero in the starplot of cells with high migratory noise and low deposition rate, but in fact it was not possible to get any value for these metrics.

***Quantifying Fractal dimension***

For quantification of fractal dimension of the matrix, we took snapshots of the matrix at the end of simulations. We then split images into RGB channels and worked with the red channel images. We converted the images to 32-bit images, applied a Gaussian filter $(\sigma=5)$, reset the colors on the Brightness and Contrast function on a non-aligned matrix and then adjusted the contrast to remove lighter areas of the image. We then converted the image back to an 8-bit image and created a mask of the image using the ridge detection plugin with a line width of 20. We used the plugin Anamorf^53^ on these masks, with a minimum branch length of 10 pixels (16.8$\mu m$) to measure the box counting fractal dimension of the entire image.
