# Supplementary material for: Matrix feedback enables diverse higher-order patterning of the extracellular matrix
Source: PLoS Comput Biol. 2019 Oct 28;15(10):e1007251. doi: 10.1371/journal.pcbi.1007251 (PMC6816557; doi:10.1371/journal.pcbi.1007251)
Supplement: S3 Text — (DOCX) [file pcbi.1007251.s013.docx]

**TextS3 Exploring parameter space**

***Principal component analysis***

We used the pcaMethods package in R, with the nipals methods to account for missing data entries, for example when the matrix was very diffuse and had no mask no measurement for curvature could be obtained. We performed PCA using the covariance matrix across all the metrics: LRA, SRA, HDM, curvature and fractal dimension.

***Varying cell-cell guidance***

We varied just cell-cell guidance and individual migratory noise, fixing matrix feedback at zero. The top row of Supplementary Fig 3a shows that in the absence of cell-cell guidance only isotropic matrix is generated, regardless of the level of individual migratory noise. Increasing the cell-cell guidance term leads to the formation of both anisotropic matrix and a spatially non-uniform distribution of both matrix and cells (Supplementary Fig 3).

***Varying matrix guidance***

Pairwise analysis of the metrics revealed that the addition of matrix feedback could produce patterns in new areas of metric-space (Supplementary Fig 4a). Line graphs show how the metrics vary as a function of increasing matrix feedback (Supplementary Fig 4b). Similarly, for simulations run at sub-confluence, PCA and pairwise analysis show that matrix feedback enhanced diversity of patterns (Fig 4c). N=20 simulations per point in parameter space were run with 800 cells in each.

***Sensitivity analysis***

Sensitivity analysis indicated that matrix diversity is robust to changes in aspect ratio^32^ (Supplementary Fig 5a), with only a modest decrease in HDM and small increase in fractal dimension for the extreme case of fibroblasts lacking any elongated morphology (aspect ratio = 1). For subsequent analysis, we chose to use an aspect ratio of 3:1 reflecting typical values measured in fibroblasts^5^.

We excluded the possibility that curvature of matrix was caused by the number of grid points comprising the matrix or the number of bins at each grid point by running a subset of simulations with finer grid points and more bins (Supplementary Fig 5b). For simulations in Fig 3 there were 128x128 grid points, as explained in text S1. For simulations in Figs 4 and 5 there were 256x256 grid points, so that one grid point has an area of approximately one quarter of the cell head. Increasing the number of grid points increases the precision of the matrix organization and deposition (Supplementary Fig 5b). This comes at a cost of increased computational time. Further, as the number of grid points is increased, average fiber density per grid point will decrease and time step will have to be reduced to avoid cells jumping over many grid points in one time step. The number of bins per grid point does not alter the ability for curvature to occur. There is no discernible difference between simulations with 8 or 40 bins per grid point (Supplementary Fig 5b). Again, as the number of bins increases, average fiber density per bin will decrease. At an extreme, if there were only two bins per grid point, the matrix would be limited to a basket-weave pattern as fibers would only be oriented north-south or east-west.

Given the two-way feedback between fibroblast orientation and matrix fibers, altering cell speed can cause small variations in matrix pattern. If cell speed is very high then fibroblasts will jump across multiple grid points in one time step, creating a dashed line of fibers. Conversely, if cell speed is very slow, then a cell may deposit fibers in the same grid point over several time points, leading to thicker matrix
