# Supplementary material for: Matrix feedback enables diverse higher-order patterning of the extracellular matrix
Source: PLoS Comput Biol. 2019 Oct 28;15(10):e1007251. doi: 10.1371/journal.pcbi.1007251 (PMC6816557; doi:10.1371/journal.pcbi.1007251)
Supplement: S4 Text — (DOCX) [file pcbi.1007251.s014.docx]

**Text S4 Fibroblast organization of the matrix affects pattern formation**

We first looked at deposition rate by fixing the rearrangement rate to zero, degradation rate to 1 and considered four different conditions: Cells with low migratory noise and low fiber deposition rate, rate set to 2 (Supplementary Fig 6a light orange circle), low migratory noise and high fiber deposition rate, rate set to 10 (Supplementary Fig 6a dark orange circle), high migratory noise and low fiber deposition rate, rate set to 2 (Supplementary Fig 6a light blue circle), high migratory noise and high fiber deposition rate, rate set to 10 (Supplementary Fig 6a dark blue circle). Example matrix produced under these four conditions is shown in Supplementary Fig 6b with starplots characterizing the different matrix patterns.

We then investigated the effects of altering the rate at which fibroblasts could reorganize fibers. We fixed the deposition rate to 1, degradation rate to zero and considered four different conditions: Cells with low migratory noise and low fiber rearrangement rate, rate set to zero (Supplementary Fig 6c light orange circle), low migratory noise and high fiber rearrangement rate, rate set to 10 (Supplementary Fig 6c dark orange circle), high migratory noise and low fiber rearrangement rate, rate set to zero (Supplementary Fig 6c light blue circle), high migratory noise and high fiber rearrangement rate, rate set to 10 (Supplementary Fig 6c dark blue circle). Example matrix produced under these four conditions is shown in Supplementary Fig 6d with starplots characterizing the different matrix patterns.

The matrix images produced *in silico* show the density of the most recently chosen bin at each grid point. Cells with low migratory noise produce thicker bundles of fibers deposited in the same bin, despite having the same overall fiber deposition rate as cells with high migratory noise (Supplementary Fig 6b and 6d, yellow and light blue boxes). Analysis in Supplementary Fig 6 is run with low matrix feedback $(w_{m}=0.04)$. Identical analysis for high matrix feedback $(w_{m}=0.2)$ is shown in Supplementary Fig 7.
