# Supplementary material for: Matrix feedback enables diverse higher-order patterning of the extracellular matrix
Source: PLoS Comput Biol. 2019 Oct 28;15(10):e1007251. doi: 10.1371/journal.pcbi.1007251 (PMC6816557; doi:10.1371/journal.pcbi.1007251)
Supplement: S5 Text — (DOCX) [file pcbi.1007251.s015.docx]

**Text S5 Quantification and normalization**

***Raw values of metrics and normalization for starplots***

For experimental and computational assays in Figure 4, short-range alignment was computed at 100$\mu m$ and long-range alignment was computed at 200$\mu m$. Otherwise, for other simulations, short-range alignment is computed as 0-200$\mu m$ and long-range alignment is computed as 200$\mu m-800\mu m$. The raw values for the metrics are given in Table 1 and Table 2

***Table 1: experimental matrix***

| Image | LRA | SRA | HDM | Curv | Frac |
| --- | --- | --- | --- | --- | --- |
| Fig 5a, Dermis | 0 | 0.14 | 1 | 40 | 1.549 |
| Fig 5a, Liver | 0 | 0.12 | 0.54 | 47 | 1.554 |
| Fig 5a, Spleen | 0 | 0.13 | 0.98 | 44 | 1.571 |
| Fig 5a, Stomach | 0.38 | 0.68 | 0.91 | 23 | 1.42 |
|  |  |  |  |  |  |
| Supplementary Fig 9b, Young dermis | 0 | 0.2 | 1 | 39 | 1.279 |
| Supplementary Fig 9b, Old dermis | 0 | 0 | 0.73 | 57 | 1.469 |
|  |  |  |  |  |  |
| Fig 9d, Normal skin | 0.28 | 0.19 | 0.73 | 43 | 1.528 |
| Fig 9d, Melanoma border | 0.68 | 0.69 | 0.73 | 43 | 1.52 |
| Fig 9d, Pre-therapy | 0.28 | 0.2 | 0.67 | 46 | 1.508 |
| Fig 9d, After therapy/resistance | 0.43 | 0.39 | 0.71 | 31 | 1.479 |

***Table 2: simulation matrix***

| Image | LRA | SRA | HDM | Curv | Frac |
| --- | --- | --- | --- | --- | --- |
| Fig 4a, red (A) | 0.67 | 0.86 | 0.19 | 18 | 1.345 |
| Fig 4a, blue (N) | 0 | 0.22 | 0 | 31 | 1.437 |
| Fig 4a, yellow (S) | 0.08 | 0.69 | 0.27 | 29 | 1.246 |
| Fig 4a, green (D) | 0.13 | 0.68 | 0.21 | 30 | 1.28 |
|  |  |  |  |  |  |
| Fig 5c, Dermis | 0 | 0.11 | 0.32 | 37 | 1.434 |
| Fig 5c, Liver | 0 | 0.16 | 0.02 | 37 | 1.411 |
| Fig 5c, Spleen | 0.15 | 0.19 | 0.27 | 34 | 1.458 |
| Fig 5c, Stomach | 0.99 | 0.99 | 0.25 | 14 | 1.412 |
|  |  |  |  |  |  |
| Supplementary Fig 6b, noBins = 8, grid points = 128 | 0.23 | 0.82 | 0.36 | 26 | 1.307 |
| Supplementary Fig 6b, noBins = 8, grid points = 256 | 0.36 | 0.88 | 0.23 | 25 | 1.253 |
| Supplementary Fig 6b, noBins = 40, grid points = 128 | 0.34 | 0.82 | 0.12 | 25 | 1.258 |
| Supplementary Fig 6b, noBins = 40, grid points = 256 | 0.3 | 0.85 | 0.07 | 19 | 1.176 |
|  | LRA | SRA | HDM | Curv | Frac |
| *Supplementary Fig 7b (yellow)* | *0.55* | *0.9* | *0.21* | *13* | *1.165* |
| *Supplementary Fig 7b (orange)* | *0.46* | *0.89* | *0.91* | *30* | *1.111* |
| *Supplementary Fig 7b (light blue)* | *0* | *0.24* | *0* | *NA* | *NA* |
| *Supplementary Fig 7b (dark blue)* | *0.27* | *0.62* | *0.99* | *32* | *1.216* |
| Supplementary Fig 7d (yellow) | 0.48 | 0.87 | 0.2 | 18 | 1.259 |
| Supplementary Fig 7d (orange) | 0.48 | 0.82 | 0.39 | 20 | 1.242 |
| Supplementary Fig 7d (light blue) | 0.04 | 0.26 | 0 | 35 | 1.181 |
| Supplementary Fig 7d (dark blue) | 0.11 | 0.38 | 0.4 | 35 | 1.179 |
|  |  |  |  |  |  |
|  |  |  |  |  |  |
| *Supplementary Fig 8b (yellow)* | *0.32* | *0.87* | *0.27* | *14* | *1.203* |
| *Supplementary Fig 8b (orange)* | *0.13* | *0.72* | *0.82* | *33* | *1.132* |
| *Supplementary Fig 8b (light blue)* | *0.02* | *0.24* | *0* | *NA* | *NA* |
| *Supplementary Fig 8b (dark blue)* | *0.17* | *0.73* | *0.95* | *30* | *1.066* |
| Supplementary Fig 8d (yellow) | 0.26 | 0.82 | 0.24 | 19 | 1.212 |
| Supplementary Fig 8d (orange) | 0.24 | 0.8 | 0.41 | 23 | 1.183 |
| Supplementary Fig 8d (light blue) | 0.25 | 0.63 | 0.01 | 25 | 1.28 |
| Supplementary Fig 8d (dark blue) | 0.27 | 0.79 | 0.5 | 30 | 1.196 |

Values in the starplots are normalized. For the experimental matrix, the following normalization functions are used:

| Metric | Normalization function for starplot vizualization |
| --- | --- |
| HDM | $\frac{x-0.5}{0.5}$ |
| Curv | $\frac{x-20}{40}$ |
| Frac | $\frac{x-1.2}{0.4}$ |

For the simulation matrix in Fig 3 the following normalization functions are used:

| Metric | Normalization function for starplot vizualization |
| --- | --- |
| HDM | $\frac{x}{0.4}$ |
| Curv | $\frac{x-10}{30}$ |
| Frac | $\frac{x-1.05}{0.4}$ |

The exception to this normalization is for starplots showing variation in degradation rate where HDM is not normalized, since now the HDM values vary between 0 and 1. These are indicated in italics in Table 2. Different normalization functions for experimental and simulation matrix are used because the real fibers and simulated fibers do not look the same, for example, the experimental fibers are generally brighter, resulting in higher HDM. NAs are given when there were not sufficient structures in the image to derive curvature or fractal dimension. In the starplots these are given a value of zero.

For the simulation matrix in Fig 4d the following normalization functions are used:

| Metric | Normalization function for starplot vizualization |
| --- | --- |
| LRA | $max(0,x-0.6)$ |
| SRA | $max(0,x-0.3)$ |
| HDM | $\frac{x}{0.35}$ |
| Curv | $\frac{x-12}{30}$ |
| Frac | $\frac{x-1.36}{0.1}$ |

***Generating matrix in Fig 5c***

The primary differences noise $(\eta)$, cell-cell guidance $\left( w_{c} \right)$ and matrix feedback $\left( w_{m} \right)$parameters in generating *in silico* matrix that mimicked the *in vivo* matrix in Fig 5a are listed in Table 1 in the main text. In addition, simulations were run for three times longer and cell speed and fiber deposition rate is variable between matrix types. These alterations reflect the gradual development of these tissues over long time periods. In other simulations in the manuscript, unless otherwise indicated, the number of matrix grid points is 128x128, equating one grid point to the approximate size of a cell head. The matrix grid in these simulations twice as fine was used to enable more precision in fiber placement. N=200 cells were used, in line with *in vivo* fibroblast density.

|  | Dermis | Liver | Spleen | Stomach |
| --- | --- | --- | --- | --- |
| Noise $(\eta)$ | 0.1 | 0.1 | 0.01 | 0.0 |
| Cell-cell guidance $\left( w_{c} \right)$ | 0.03 | 0.03 | 0 | 0.03 |
| Matrix feedback $\left( w_{m} \right)$ | 0.4 | 0.8 | 0.01 | 0 |
| Deposition rate | 2 | 1 | 2 | 2 |
| Mean Speed $(\mu/h)$ | 0.7 | 0.7 | 1.8 | 3.5 |
| Speed standard deviation $(\mu/h)$ | 0.1 | 0.1 | 0.3 | 0.5 |
| Number of matrix grid points | ${256}^{2}$ | ${256}^{2}$ | ${256}^{2}$ | ${256}^{2}$ |
