# Supplementary material for: Matrix feedback enables diverse higher-order patterning of the extracellular matrix
Source: PLoS Comput Biol. 2019 Oct 28;15(10):e1007251. doi: 10.1371/journal.pcbi.1007251 (PMC6816557; doi:10.1371/journal.pcbi.1007251)
Supplement: S6 Text — (DOCX) [file pcbi.1007251.s016.docx]

**Text S6 Choosing parameters for mimicking *in vivo* tissues**

In Park et al. 2019, we show that *in vitro*, non-aligning fibroblasts moving on glass in sub-confluence have noise attuned to, $\eta=0.18$. From this, we estimate that reasonable values for noise would be in the range $\eta\in[0, 0.2]$. In the same manuscript, we fitted the value of cell-cell guidance for the aligning fibroblasts to be $w_{c}\approx0.05$. From this, we estimate that a reasonable search space for cell-cell guidance would be in the range $w_{c}\in\left[ 0,0.1 \right].$ In this manuscript, the experiment shown in Figure 2a enabled us to fit matrix feedback, which we estimate to be $w_{m}\approx0.27$, as described in the methods section. This level of matrix feedback is in two dimensions, whereas in three dimensions matrix feedback is likely to be higher given the apical and basal constraints that matrix fibers can provide to cells. Therefore, we did not restrict our search on matrix feedback and searched in the range $w_{m}\in[0,1]$. We varied noise and cell-cell guidance by increments of 0.01 for their specified ranges, and varied matrix feedback by increments of 0.01 in the range [0,0.1] and by increments of 0.1 in the range [0.1,1]. We stepped through parameter space by varying one parameter at a time and fixing all the other parameters. In this way, we were able to get a comprehensive view of parameter space.

We then quantified the four experimental *in vivo* matrix patterns (Figure 5a, 5b) according to the five metrics. The raw values of these measurements are as follows:

| Image | LRA | SRA | HDM | Curv | Frac |
| --- | --- | --- | --- | --- | --- |
| Fig 5a, Dermis | 0 | 0.14 | 1 | 40 | 1.549 |
| Fig 5a, Liver | 0 | 0.12 | 0.54 | 47 | 1.554 |
| Fig 5a, Spleen | 0 | 0.13 | 0.98 | 44 | 1.571 |
| Fig 5a, Stomach | 0.38 | 0.68 | 0.91 | 23 | 1.42 |

These values are listed in Text S5. An important challenge arises from these values: there are discrepancies between the *in vivo* and *in silico* metrics. The stomach matrix *in vivo* looks highly aligned, and yet has LRA=0.38 and SRA=0.68. An *in silico* matrix that looks similarly aligned would have much higher LRA and SRA, closer to one. This is because *in silico*, the output fiber orientations are recorded exactly, whereas *in vivo* the images are noisier with variation in fiber intensity. Whilst the theoretical upper limit for both LRA and SRA is 1, the upper limit *in vivo* is probably closer to the observed values of 0.38 and 0.68 respectively. Similarly, the mean value of HDM *in silico* through all parameter space is 0.3, and very few simulations have HDM higher than 0.5 whilst Frac *in silico* is also consistently lower than the four *in vivo* images. This is most likely due to differences in fiber intensity as picked up by the microscope, fiber thickness and duration over which the matrix patterns develop *in vivo*. Whilst the matrix patterns *in silico* qualitatively look very similar to *in vivo* patterns, there is a gap between the *in vivo* and *in silico* values due to the nature of how the matrix patterns are captured. It is difficult to ascertain an appropriate function for

“inter”-translation from *in silico* to *in vivo* values with just four *in vivo* images, making a Bayesian inference or similar approach challenging.

There are however a number of heuristic arguments we were able to make from the *in vivo* patterns through which we can manually pair matrix patterns. Firstly, the stomach is highly aligned. From stepping through parameter space, we know that the area of parameter space corresponding to high LRA and SRA is where noise and matrix feedback are low, and cell-cell guidance is non-zero. Secondly, the *in vivo* liver is highly corralled which we know occurs in the region of parameter space where matrix feedback is high. Thirdly, spleen matrix appears to be made up of long straight fibers (suggesting low individual migratory noise) which are uncoordinated (suggesting low cell-cell guidance) and independent (suggesting low matrix feedback). Finally, the dermis matrix is swirl-like, diffuse and reinforced, which we know corresponds to the region of parameter space where matrix feedback and noise are high.

Through this line of argument, we were able to choose the *in silico* patterns shown in Figure 5c. Qualitatively they agree well with the *in vivo* images. Encouragingly, whilst the raw measurements for the five metrics are different from the *in vivo* values for the reasons described above, the “intra”-comparison between the four patterns is consistent with the *in vivo* “intra”-comparisons. For example, the HDM of the liver both *in vivo* and *in silico* is the lowest of the four, the Frac and Curv of the stomach is the lowest of the four and the Frac of the spleen is the highest. This suggests that we have correctly identified that areas in parameter space which produce the correct patterns relative to the system (*in silico* vs *in vivo*). In the future, with access to far more experimental images, deep-learning approaches could provide a more systematic pairing between *in silico* and *in vivo*.
