# Supplementary material for: Matrix feedback enables diverse higher-order patterning of the extracellular matrix
Source: PLoS Comput Biol. 2019 Oct 28;15(10):e1007251. doi: 10.1371/journal.pcbi.1007251 (PMC6816557; doi:10.1371/journal.pcbi.1007251)
Supplement: S7 Text — (DOCX) [file pcbi.1007251.s017.docx]

**Text S7 Experimental methods**

***Cell lines and reagents***

Human fibroblasts were isolated from patient tissues of vulval (VCAF8) and immortalized with lentiviral HTERT as described in Gaggioli, C., et al. All patient samples were collected under ethical approval 10/H0304/14 and 15/EE/0151. Cells were selected using 400 μg mL-1 hygromycin and maintained in DMEM (Invitrogen), 10% FCS (PAA Labs), 1% ITS (insulin–transferrin–selenium; #41400-045; Invitrogen) supplement.

Mouse fibroblasts (CAF1) were isolated from transgenic FVB/n mice expressing the Polyoma Middle T antigen oncogene under the Mouse Mammary Tumor Virus promoter (MMTV-PyMT) as described in Calvo, F., et al. Cells were immortalized with HPV-E6 retrovirus, selected using 2.5 μg mL-1 puromycin and maintained in DMEM, 10% FCS and 1% ITS as above.

For cell tracking experiments fibroblasts were infected with the retroviral nuclear tag AcGFP-NLS (pLNCX2) and selected using 500 μg mL-1 geneticin. The functional upstream domain (FUD) of adhesin F1 of *Streptococcus pyogene* was used to disrupt fibronectin assembly and was kindly provided by C. Albiges-Rizo (IAB, Grenoble, France).

***Fibroblast derived matrix assay***

The fibroblast derived matrix assay was performed as described in Franco-Barraza, J., et al. Briefly, 24 well glass bottom MatTek dishes (P35-1.5-14-C, MatTek Co., Ashland, MA, USA) were pre-prepared with 0.2% gelatin solution for 1 hr at 37 °C, followed by 1% glutaraldehyde for 30 min at room temperature. The plate was washed twice with PBS then incubated with 1M ethanolamine for 30 min at room temperature. The plate was washed twice with PBS before seeding 7 x 10^4^ cells in media supplemented with 100μg/ml ascorbic acid ((+)-Sodium L-ascorbate, A4034, Sigma). The cells were maintained for 6 days and the media changed every two days. Cells were removed using the extraction buffer described and washed several times with PBS before undertaking immunofluorescence for ECM components. The ECM was stained with the anti-fibronectin antibody (1:1000 dilution, Sigma, F3648) or anti-fibronectin-FITC (1:50 dilution: Abcam, ab72686). Where indicated 7 x 10^3^ fibroblasts (sub-confluent) or 7 x 10^4^ cells (confluent) were plated on top of pre-existing matrices for time-lapse imaging.

***Time-lapse microscopy for persistence analysis***

Nuclear labelled fibroblasts were seeded at approx. 7 x 10^3^ cells per well in 24 well glass bottom MatTek dish and imaged approximately 8 hrs later. Bright-field and epifluorescence time-lapse imaging was performed at 37 °C and 5% CO_2_ with an inverted microscope (Nikon Ti2 inverted microscope fitted with a Okolab environmental chamber and CO2 mixer). Bright-field and epifluorescence images were taken every 10 min through a ×10 PlanFluor, NA 0.3 Ph1, Nikon objective. The imaging system includes a SpectraX LED light engine (Lumencor) fitted with standard filters and Photometrics Prime scientific CMOS camera. The microscope was managed using Micro-Manager v2.0 software.

Where indicated cells were pre-treated with 500nM FUD and the media changed every 24 hrs. Cells tracked using the ImageJ Trackmate plug in. Persistence was calculated as the ratio of shortest linear distance between two points of migration (displacement) to the total distance traversed by the cell (distance) over 16 hr intervals.

***Immunohistochemistry of breast cancer microarray***

Human invasive ductal and lobular breast carcinoma microarray was stained for picrosirus red.

***In vivo imaging of collagen***

The organs of platelet derived growth factor receptor, alpha polypeptide; targeted mutation 11 (MGI:2663656) with nuclear labelled EGFP were imaged using second harmonic confocal microscopy. Supplementary Fig 8a shows a subsection of stomach dermis together with an *in silico* representation of a similar system. Imaging of this kind shows how matrix can change over time, for instance in ageing (Supplementary Fig 8b and 8c). Supplementary Fig 8d shows the complementary Gomori Trichome imaging of Fig 4d.

***Fibroblast derived matrix assay***

The fibroblast derived matrix assay was performed as described in Franco-Barraza, J., et al. Briefly, 24 well glass bottom MatTek dishes (P35-1.5-14-C, MatTek Co., Ashland, MA, USA) were pre-prepared with 0.2% gelatin solution for 1 hr at 37 °C, followed by 1% glutaraldehyde for 30 min at room temperature. The plate was washed twice with PBS then incubated with 1M ethanolamine for 30 min at room temperature. The plate was washed twice with PBS before seeding 7 x 10^4^ cells in media supplemented with 100μg/ml ascorbic acid ((+)-Sodium L-ascorbate, A4034, Sigma). The cells were maintained for 6 days and the media changed every two days. Cells were removed using the extraction buffer described and washed several times with PBS before undertaking immunofluorescence for ECM components. The ECM was stained with the anti-fibronectin antibody (1:1000 dilution, Sigma, F3648) or anti-fibronectin-FITC (1:50 dilution: Abcam, ab72686). Where indicated 7 x 10^3^ fibroblasts (sub-confluent) or 7 x 10^4^ cells (confluent) were plated on top of pre-existing matrices for time-lapse imaging.
